# Blood Coagulation and Beyond: Position Paper from the Fourth Maastricht Consensus Conference on Thrombosis

**DOI:** 10.1055/a-2052-9175

**Published:** 2023-05-12

**Authors:** Asim Cengiz Akbulut, Ryanne A. Arisz, Constance C. F. M. J. Baaten, Gaukhar Baidildinova, Aarazo Barakzie, Rupert Bauersachs, Jur ten Berg, Wout W. A. van den Broek, H. C. de Boer, Amandine Bonifay, Vanessa Bröker, Richard J. Buka, Hugo ten Cate, Arina J. ten Cate-Hoek, S. Cointe, Ciro De Luca, Ilaria De Simone, Rocio Vacik Diaz, Françoise Dignat-George, Kathleen Freson, Giulia Gazzaniga, Eric C. M. van Gorp, Anxhela Habibi, Yvonne M. C. Henskens, Aaron F. J. Iding, Abdullah Khan, Gijsje H. Koenderink, Akhil Konkoth, Romaric Lacroix, Trisha Lahiri, Wilbur Lam, Rachel E. Lamerton, Roberto Lorusso, Qi Luo, Coen Maas, Owen J. T. McCarty, Paola E. J. van der Meijden, Joost C. M. Meijers, Adarsh K. Mohapatra, Neta Nevo, Alejandro Pallares Robles, Philippe Poncelet, Christoph Reinhardt, Wolfram Ruf, Ronald Saraswat, Claudia Schönichen, Roger Schutgens, Paolo Simioni, Stefano Spada, Henri M. H. Spronk, Karlygash Tazhibayeva, Jecko Thachil, Rocio Vacik Diaz, L. Vallier, Alicia Veninga, Peter Verhamme, Chantal Visser, Steve P. Watson, Philip Wenzel, Ruth A. L. Willems, Anne Willers, Pengyu Zhang, Konstantinos Zifkos, Anton Jan van Zonneveld

**Affiliations:** 1 Department of Biochemistry, Cardiovascular Research Institute Maastricht, Maastricht University, Maastricht, The Netherlands; 2 Department of Hematology, Erasmus MC, University Medical Center Rotterdam, Rotterdam, The Netherlands; 3 Institute for Molecular Cardiovascular Research (IMCAR), University Hospital RWTH Aachen, Aachen, Germany; 4 Center for Thrombosis and Hemostasis (CTH), University Medical Center Mainz, Johannes Gutenberg-University Mainz, German Center for Cardiovascular Research (DZHK), Partner Site Rhein-Main, Mainz, Germany; 5 Cardioangiologisches Centrum Bethanien – CCB, Gefäß-Centrum, Frankfurt am Main, Germany; 6 Department of Cardio-Thoracic Surgery, Maastricht University Medical Center, Maastricht, The Netherlands; 7 Department of Cardiology, St. Antonius Hospital, Nieuwegein, The Netherlands; 8 Department of Internal Medicine (Nephrology) and the Einthoven Laboratory for Vascular and Regenerative Medicine, Leiden University Medical Center, Leiden, The Netherlands; 9 Aix-Marseille University, C2VN, INSERM 1263, INRAE 1260, Marseille, France; 10 Department of Hematology and Vascular Biology, CHU La TIMONE, APHM, Marseille, France; 11 Institute of Cardiovascular Sciences, College of Medical and Dental Sciences, University of Birmingham, Vincent Drive, Birmingham, United Kingdom; 12 Thrombosis Expertise Center, Maastricht University Medical Center, Maastricht, The Netherlands; 13 Laboratory of Morphology of Neuronal Networks and Systems Biology, Department of Mental and Physical Health and Preventive Medicine, University of Campania “Luigi Vanvitelli,” Napoli, Italy; 14 Center for Molecular and Vascular Biology, Department of Cardiovascular Sciences, KU Leuven, Leuven, Belgium; 15 Department of Clinical-Surgical, Diagnostic and Pediatric Science, University of Pavia, Pavia, Italy; 16 Department of Viroscience, Erasmus Medical Center, Rotterdam, The Netherlands; 17 Central Diagnostic Laboratory, Unit for Hemostasis and Transfusion, Maastricht University Medical Centre + , Maastricht, The Netherlands; 18 MRC Weatherall Institute of Molecular Medicine, Radcliffe Department of Medicine and National Institute of Health Research (NIHR) Oxford Biomedical Research Centre, University of Oxford, Oxford, United Kingdom; 19 Department of Bionanoscience, Kavli Institute of Nanoscience Delft, Delft University of Technology, Delft, The Netherlands; 20 Wallace H. Coulter Department of Biomedical Engineering, Georgia Institute of Technology and Emory University, Atlanta, Georgia, United States; 21 CDL Research, University Medical Center Utrecht, Utrecht University, Utrecht, The Netherlands; 22 Department of Biomedical Engineering, Oregon Health and Science University, Portland, Oregon, United States; 23 Department of Molecular Hematology, Sanquin Research, Amsterdam, The Netherlands; 24 Department of Experimental Vascular Medicine, Amsterdam UMC, University of Amsterdam, Amsterdam, The Netherlands; 25 Amsterdam Cardiovascular Sciences, Pulmonary Hypertension and Thrombosis, Amsterdam, The Netherlands; 26 Department of Immunology, Weizmann Institute of Science, Rehovot, Israel; 27 Department of Immunology, Faculty of Medicine, Technion – Israel Institute of Technology, Haifa, Israel; 28 R and T Department, BioCytex, Marseille, France; 29 Center for Benign Haematology, Thrombosis and Haemostasis, Van Creveldkliniek, UMC Utrecht, University Utrecht, Utrecht, The Netherlands; 30 General Internal Medicine and Thrombotic and Hemorrhagic Diseases Unit, Department of Medicine, Padua University Hospital and University of Padua Medical School, Padua, Italy; 31 Cancer Center of Shymkent, Al-Farabi Kazakh National University, Almaty, Kazakhstan; 32 Department of Haematology, Manchester University Hospitals, Manchester, United Kingdom; 33 Department of Cardiovascular Sciences, University of Leuven, Leuven, Belgium; 34 Leibniz Institute for Analytical Sciences - ISAS-e.V., Dortmund, Germany

**Keywords:** antiplatelet agent, artificial surfaces, atherosclerosis, oral anticoagulants, thrombosis

## Abstract

The Fourth Maastricht Consensus Conference on Thrombosis included the following themes. Theme 1: The “coagulome” as a critical driver of cardiovascular disease. Blood coagulation proteins also play divergent roles in biology and pathophysiology, related to specific organs, including brain, heart, bone marrow, and kidney. Four investigators shared their views on these organ-specific topics. Theme 2: Novel mechanisms of thrombosis. Mechanisms linking factor XII to fibrin, including their structural and physical properties, contribute to thrombosis, which is also affected by variation in microbiome status. Virus infection-associated coagulopathies perturb the hemostatic balance resulting in thrombosis and/or bleeding. Theme 3: How to limit bleeding risks: insights from translational studies. This theme included state-of-the-art methodology for exploring the contribution of genetic determinants of a bleeding diathesis; determination of polymorphisms in genes that control the rate of metabolism by the liver of P2Y12 inhibitors, to improve safety of antithrombotic therapy. Novel reversal agents for direct oral anticoagulants are discussed. Theme 4: Hemostasis in extracorporeal systems: the value and limitations of ex vivo models. Perfusion flow chamber and nanotechnology developments are developed for studying bleeding and thrombosis tendencies. Vascularized organoids are utilized for disease modeling and drug development studies. Strategies for tackling extracorporeal membrane oxygenation-associated coagulopathy are discussed. Theme 5: Clinical dilemmas in thrombosis and antithrombotic management. Plenary presentations addressed controversial areas, i.e., thrombophilia testing, thrombosis risk assessment in hemophilia, novel antiplatelet strategies, and clinically tested factor XI(a) inhibitors, both possibly with reduced bleeding risk. Finally, COVID-19-associated coagulopathy is revisited.

## Graphical abstract

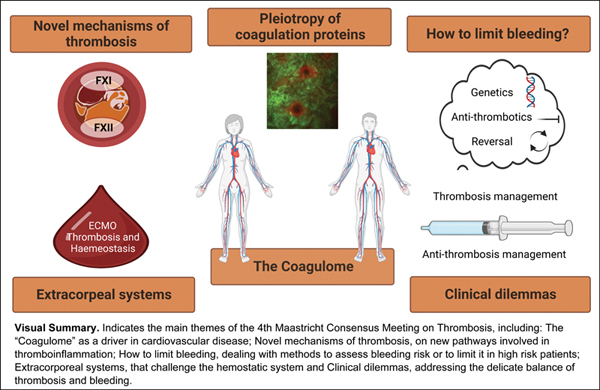

## Introduction

During the Fourth Maastricht Consensus Conference on Thrombosis (MCCT), held in April 2022, the main theme of the conference was “Blood coagulation and beyond” expressing the desire of the organizers to look beyond boundaries. A characteristic of this conference is the strong interaction among presenters and audience encouraged by the breakout sessions following presentations creating room for in-depth discussions among basic, translational, and clinical scientists from different backgrounds. The MCCT meeting focused on five different topics, to be addressed below. The authors comprise not only faculty but also PhD students that were actively involved in discussions as well as note taking of the discussion sessions; these notes and the summary of the presentations provided the basis for this article in which all actively involved act as contributory authors. This meeting was co-organized with the EU-Marie Curie International Training Network TICARDIO and all PhD students from this network were contributing to this article.

## Theme 1: The “Coagulome” as a Critical Driver of Cardiovascular Disease

### The Brain Coagulome

To briefly introduce the term coagulome, which we use in analogy to the previously used term “endotheliome” to describe a multifactorial approach to the endothelium, 1 assessing its multifunctional properties in health and disease by combining different methods, to obtain an integrated image of this pivotal cellular compartment, is essential.

Primary and secondary prevention of ischemic stroke (IS) benefits from antiplatelet and anticoagulant therapies. 2 However, compared with coronary heart diseases (CHDs), P2Y12 inhibitors other than clopidogrel have no clinical use in primary stroke prevention and can be contraindicated (prasugrel) in patients with a previous stroke for increased risk of intracranial bleeding. 3 Similarly, dual antiplatelet therapy (DAPT) is effective just in the early phases of the IS (21–30 days) before becoming useless or detrimental. In the case of vorapaxar, which is the only approved drug of a novel class antithrombotic agent acting on the protease-activated receptor-1 (PAR-1), trials directly assessing stroke management are lacking. 4 PAR-1 is fundamental for pleiotropy of coagulation factors in the central nervous system (CNS). 5 The main proteases that can activate PAR-1 are matrix metalloproteinase 9 (MMP-9) 5 and thrombin, whose activation state, concentration, and association with activated protein C (aPC) lead to differential pathway activation in physiology 6 as well as CNS pathologies.

Factor (F) XI has been shown to be involved in thrombus stabilization during stroke. 7 In a large population, elevated FXI was associated with the risk of IS and a FXI:C level <15 U/dL incurred protection against stroke. 8 9 The FXIa level was higher in subjects with previous stroke compared with those with a history of transient ischemic attack (TIA) (34 vs. 11.4%, *p* < 0.0001), suggesting that FXIa is associated with worse functional outcomes of cerebrovascular disease. 10 The related mechanism could be that inhibition of FXI(a) reduces thrombin generation, activation of TAFI (thrombin activatable fibrinolysis inhibitor), and ultimately may enhance the lysis of clots that form or embolize into cerebral arteries. 11

Consistent with those findings in human studies, in the mouse model of acute IS (temporal occlusion of the middle cerebral artery), administration of antibody 14E11 that blocks the activated FXII (FXIIa)-dependent activation of FXI resulted in a significant reduction in infarct size and a significant improvement in neurological function compared with the control group. 12 Clinical and experimental evidence demonstrated that coagulation proteins have pleiotropic effects on the CNS not limited to physiological repair of vascular damage and pathological ischemic/hemorrhagic stroke.

The different effects of antiplatelet and anticoagulant agents on the CNS can be in part due to the existence of a unique and complex interface represented by the neurovascular unit (NVU). Indeed, other organs can promptly differentiate their own blood vessels when repairing a lesion, or for metabolic reasons even without perturbing the tissue integrity, the same cannot be said for the CNS. The NVU is a unique integrated frontier in which the mesenchymal cells (endothelial cells [ECs], pericytes, smooth muscle cells, fibroblasts) do not originate from within the CNS tissue (purely ectodermal formed by neurons and macroglia) but penetrate without violating its integrity during embryogenesis. During CNS development through a clear contribution of coagulation factors such as tissue factor pathway inhibitor (TFPI), FV, FVII, and FX, the mesenchyma enters the nervous parenchyma. 13 The same happens for the resident immune cells, the microglia, which is a distinct population of myeloid cells, not differentiated from the bone marrow (BM), but originating from the yolk sac. 14 Hence the coagulation factors, as mentioned, do not limit their intervention to vascular repair and exert their function also on the nervous tissue, justifying their emerging role in neurological diseases other than stroke.

This pleiotropy has been demonstrated in various pathologies that have no strict vascular etiology, such as multiple sclerosis (MS), Parkinson's disease (PD), and Alzheimer's disease (AD). 5 TFPI was shown to be increased in the frontal cortex of AD brains compared with healthy controls. 15 In MS patients, TFPI levels were higher in the group of progressive MS compared with relapsing–remitting MS and healthy controls. Same results were obtained for plasminogen activator inhibitor-1 (PAI-1) expression in these groups. 16 In a randomized controlled trial (RCT) of recovering MS patients, it was shown that plasma levels of TFPI 17 and other coagulation inhibitors (e.g., protein S) increased with increasing recovery rate and patients with a generally low level of TFPI in earlier disease states showed better rehabilitation afterwards. 17

As a neurodegenerative disease, AD is characterized by abnormal loss of cholinergic neurons in areas of the brain that are primarily responsible for cognition and memory. The key pathological elements in AD have been proven to be amyloid-β (Aβ) peptides and neurogenic fiber tangles. In animal studies, human amyloid precursor protein (hAPP) transgenic mice from line J20 (hAPP-J20 mice) are used to establish the AD model, and the results have shown that coagulation factors are involved in the metabolism of Aβ, 18 19 which can lead to the activation of FXII, resulting in FXI activation and thrombin generation, ultimately leading to a prothrombotic environment that contributes to the development of AD. These data are supported by decreased levels of plasmatic FXI in AD patients, with depletion of its inhibitor, suggesting a chronic activation with subsequent inactivation and clearance of FXI during the disease. 20 Moreover, in the same patients, activation of the intrinsic coagulation pathway is supported by elevated plasmatic fibrin levels. 20 Compared with cognitively healthy people or patients diagnosed with mild cognitive impairment, patients who are diagnosed with AD have significantly increased plasmatic levels of FXI. 21 FXI may therefore be a predictor of AD-type diagnosis, as an increase in FXI has been associated with a reduction in cognitive function. 21 22 Proteomic analyses of plasma and postmortem brain tissues (the inferior frontal cortex, superior frontal cortex, and cerebellum) from AD patients demonstrated a clear activation of complement coagulation cascade, in particular of FXII and FXIII, further corroborating this hypothesis. 23

The pathogenesis of AD could be particularly sensitive to NVU disruption; fibrin deposition, possibly an end stage product resulting from the long-term dysfunction of the NVU, has been demonstrated in both large vessels and capillaries of AD patients and can have a great impact on metabolic coupling, particularly in the hippocampal region. 24 Parenchymal deposition of fibrin, as the last step of the coagulation cascade, could enhance the inflammatory state and contribute to the loss of integrity of the blood–brain barrier (BBB). In the dysfunctional NVU, astrocytic apolipoprotein E4 (APOE4), interacting with pericytic low-density lipoprotein receptor-related protein 1, through cyclophilin A (CypA) signaling, increases MMP-9 transduction and thrombin/PAR-1 signaling. 25 The BBB breakdown was more severe in carriers of APOE4, an identified genetic risk factor for AD with cognitive impairment, independently of AD biomarkers, both Aβ and tau. The BBB damage, measured in vivo by magnetic resonance imaging (MRI) as well as pericyte- and platelet-derived biomarkers such as soluble platelet-derived growth factor receptor β (sPDGFRβ) predicted the future cognitive status in carriers even after controlling the analysis for Aβ and tau levels. 25 These predictive biomarkers correlated with increased CypA - MMP9 activity in the cerebrospinal fluid (CSF) and are very promising for early diagnosis of AD. Fibrin–Aβ fibrils are not accessible to breakdown by plasmin, activate FXII, and inhibit microglia/macrophages scavenging through CD11b silencing. 26 Blockage of fibrin–Aβ interaction (as demonstrated through RU-505) could pave the way to overcome the failures in disease-modifying therapies for neurodegeneration. 27 Finally, FXIIa, high molecular-weight kininogen, and kallikrein activities, all thrombo-inflammatory mediators, are detected in AD and their effects can be experimentally attenuated by FXII depletion. 28

The aforementioned data reinforce the idea of the vicious circle starting with the regional failure of the NVU and leading to protein deposition and neuroinflammation.

Potential areas for future investigation:

- Investigate the emerging pleiotropic role of coagulation cascade in the CNS with the central role of PAR1 interference.
- Explore the role of pericytes for NVU stability, for vascular tone, permeability, and metabolic regulation and as early CSF biomarkers of AD.
- Search for brain-specific biomarkers of the patient's thrombo-inflammatory state to develop noninvasive, easy-to-access diagnostic/prognostic tools.
- Diffuse homogeneous protocols for the evaluation of BBB integrity using standard MRI or PET-CT (positron emission tomography-computed tomography) scans, to be correlated with novel biomarkers (e.g., sPDGFRβ) and ATN (Amyloid, Tau, Neurodegeneration) classification in clinical settings.
- Targeting the fibrin/CD11b complex and inhibiting FXIa and FXII with novel or existing drugs in future clinical trials for neurodegeneration, especially AD.

### The Cardiovascular Coagulome: Focus on Thrombin and Inhibition of Its Amplification

While the role of thrombin generation in CHD, including the process of atherogenesis and atherothrombosis, has been demonstrated in experimental and clinical studies, current research focuses on specific coagulation proteases, including FXI and the tissue factor (TF)/TFPI axis.

FXI, as a component of the intrinsic pathway of coagulation, is activated by FXIIa and then proceeds to the downstream coagulation cascade that eventually triggers thrombin generation. In addition, FXI can also be feedback-activated by thrombin, further accelerating fibrin formation. Over the past decades, many studies have attempted to investigate the role of FXI in thrombin generation and its relationship with thrombus formation.

In the animal model of atherosclerosis (ApoE knockout mice, ApoEko), knockout of FXI reduced peripheral atherosclerosis by up to 33%. 29 30 In another animal study, low-density lipoprotein receptor knockout (Ldlr ^−/−^ ) mice combined with high-fat diet were treated with anti-FXI antibody (14E11) or FXI ASO. Compared with controls, 14E11 and FXI-ASO both reduced the area of atherosclerotic lesions in the proximal aorta, and 14E11 also reduced aortic sinus lesions. 31 These data suggest that FXI plays a role in atherogenesis, and that depletion of FXI may reduce development of atherosclerosis. Another indicative factor for thrombogenicity in coronary lesions might be the co-localization of TFPI with TF. Tissue studies of coronary atherosclerotic plaques revealed expression of TFPI in ECs, macrophages, foam cells, and smooth muscle cells. Co-localization with TF only occurred in ECs and macrophages in the groups of highest severity and was also found in the necrotic lipid core. 32

CHD is the result of partial or complete occlusion of the coronary arteries due to thrombosis, which impairs the blood supply to the heart muscle. Outcomes of the PRIME study including nearly 10,000 men showed that patients with a general low free TFPI plasma concentration had a more than twofold increased risk of developing CHD. This effect was increased to sevenfold, when von Willebrand factor (vWF) levels were increased. 33 Additionally, TFPI levels were generally higher in non-ST segment elevation myocardial infarction (NSTEMI) compared with ST-segment elevation myocardial infarction (STEMI) patients. 34 In another study, TFPI levels in hospitalized patients with acute coronary syndrome (ACS) were indicative for the severity of myocardial infarction (MI) but were not associated with mortality. 35

Numerous polymorphisms of TFPI have been studied over the last decades that in part correlate with increased risk of cardiovascular disease, 36 ^,^ 37 but sometimes only shown to be related to altered TFPI plasma levels, but not to an increased risk for CHD. 38 39 40 However, studies reporting blood concentrations of coagulation should be carefully interpreted, since lower circulating levels could reflect both reduced production or increased consumption (or vice versa). This requires more research to understand the pathophysiology in the respective disease setting to improve applicability of a given coagulation factor as a putative biomarker.

Lorentz et al found that mice treated with an anti-FXI antibody, 14E11, had decreased myocardial infarct size in a model of ischemia/reperfusion (I/R) injury, indicating that FXI activation or activity might contribute to cardiac I/R injury. 41 Kossmann et al revealed that depletion of FXI could not only decrease a vascular coagulation–inflammatory circuit in angiotensin II-induced arterial hypertension, but also prevent arterial hypertension-induced end-organ damage. 42

The role of FXI in acute MI (AMI) is less clear than in stroke. Patients with lower levels of FXI are at less risk of venous thromboembolism (VTE) and MI 43 and FXI level is correlated with MI risk among men in the study of Myocardial Infarction Leiden. 44 Butenas et al reported that plasma FXIa level could be quantified in most patients with ACSs, whereas it was undetectable in age-matched healthy controls. 45 However, conflicting data exist. Salomon et al reported similar incidences of AMI in patients with severe FXI deficiency and the general population and inherited FXI deficiency seems to be not protective against AMI. 46 Results from the Risk of Arterial Thrombosis in Relation to Oral Contraceptives (RATIO) case-control study showed that high levels of FXI are associated with IS, but are not or to a lesser extent associated with MI, in young women. 47 These data suggest that the contribution of FXI in thrombosis varies between vascular beds and sex. The question why the deficiency of FXI has disparate effects on acute IS and MI, and what the exact role of FXI on MI is, still requires further exploration.

Atrial fibrillation (AF) is the most common sustained cardiac rhythm disorder and is associated with a prothrombotic state. It was shown in a cohort study that in long-term follow-up, the FXIa level in circulating blood has been associated with poor prognosis such as IS and cardiovascular death in AF patients on anticoagulants. 48 Recently, the FXIa inhibitor asundexian at two doses (20 and 50 mg daily) showed lower bleeding rates than the active comparator, the FXa inhibitor apixaban 5 mg, in a phase II trial in AF at risk for stroke. However, it still remains to be further investigated to what extent inhibition of FXI(a) is equally or more effective than established direct oral anticoagulants (DOACs) to prevent thrombotic events and if they could improve long-term prognosis of AF. 49 Current clinical studies testing the efficacy and safety of different types of FXI inhibitors, or FXI-lowering agents, is discussed further on in this article.

Potential areas for future investigation:

- A possible therapeutic target to prevent thrombo-inflammation occurring in the heart is the direct targeting of FXI or FXII that both can bind to platelets that concentrate both factors through their GPIbα and PAI-1 surface proteins and thereby increasing thrombin generation. A potential drawback of targeting in particular FXII is the increased risk for infection. Patients who are receiving FXI/FXII inhibitor treatment should therefore be monitored on a regular basis for markers of infection or inflammatory disease, such as concentration of complement fragment C1q in soluble plasma.
- It remains to be investigated in clinical trials what exactly the differences are between inhibition of FXI and FXII and whether there is any redundancy to targeting prekallikrein. Also, potential mechanisms of bypassing FIX activation should be elucidated beforehand.
- It is still unclear whether possible therapeutic options against cardiovascular thrombosis would also be suitable for treating or preventing thrombotic events in the management of aortic valve stenosis. One major risk factor for aortic valve stenosis patients is acquired vWF syndrome, which is directly related to disease severity. In this condition, vWF becomes proteolytically cleaved by high shear forces as it passes the stenotic valve. This results in a higher bleeding risk for patients of aortic valve stenosis that is not easy to measure.
- Another possible treatment strategy for preventing hypercoagulation in the heart might be drugs targeting TF or FVII, but to avoid bleeding, a safer approach is the targeting of TF signaling pathways. Also, inhibitors of TF/FVII, such as NAPc2, 50 could be repurposed as anti-inflammatory or antifibrotic drugs.

### Bone Marrow: Role of Coagulation in Cell Trafficking

Following hematopoietic stem cell transplantation (HSCT), the blood and immune system take a long time to regenerate. This period is dangerous since patients have a low ability to mount an immune response and are at a high risk for life-threatening infections and internal bleeding. Therefore, finding novel ways to shorten the recovery time will reduce morbidity and mortality rates post HSCT. Previously the role of coagulation-associated pathways in the regulation of murine hematopoietic stem and progenitor cell (HSPC) maintenance within the BM has been described. 51 52 53 54 55 Importantly, these pathways also regulate the mobilization of human HSPC in healthy stem cell donors, and moreover, impact the neutrophil and platelet engraftment rates of patients post HSCT. 56 In particular, the involvement of PAR1, the major thrombin receptor in human HSPC regulation, was shown through analysis of peripheral blood samples obtained from 20 healthy HSPC donors before and after treatment with G-CSF. Overall, the baseline levels of PAR1 expression on circulating mononuclear cells (MNCs) before G-CSF treatment positively correlated with higher yields of total G-CSF-mobilized leukocytes and CD34+ HSPC. To further assess the requirement for functional PAR1 signaling in human HSPC mobilization, chimeric immune-deficient mice were utilized, pre-engrafted with human cord blood HSPC. Importantly, blocking PAR1 signaling by in vivo administration of a specific PAR1 antagonist inhibited G-CSF-induced mobilization of human white blood cells and CD34+ HSPC to the circulation of chimeric mice. Migration, homing, engraftment, and mobilization of human HSPC are dependent on the chemokine CXCL12, which is highly expressed in the BM, and its major receptor CXCR4, which is expressed by human HSPC. Importantly, in vitro migration of human HSPC toward a gradient of the chemokine CXCL12 was inhibited by blocking PAR1, suggesting a role in human HSPC migration and engraftment. Indeed, by following recovery parameters of patients transplanted with G-CSF-mobilized cells, accelerated neutrophil and platelet engraftment in patients transplanted with mobilized cells expressing higher PAR1 levels on MNC at baseline was demonstrated. Utilizing functional preclinical murine models, the importance of the thrombin/PAR1/nitric oxide (NO) axis as a crucial regulatory pathway mediating G-CSF-induced mobilization was demonstrated. 57 The most primitive, BM retained, long-term repopulating hematopoietic stem cells (HSCs) express endothelial protein C receptor (EPCR). Its major ligand, aPC, is also produced in the BM. Signaling via the APC/EPCR/PAR1 axis controls BM HSC adhesion and retention via NO inhibition and activation of adhesion interactions. In contrast, G-CSF activates NO generation in HSPC, EPCR shedding from their surface, which leads to their mobilization. Importantly, EPCR expression is essential for chemotherapy resistance of normal mouse 53 and human HSC 58 via adhesion interactions suggesting that, unfortunately, EPCR also protects human acute myeloid leukemia stem cells from radio- and chemotherapy treatments. To conclude, Nevo and colleagues identified a new player participating in the regulation of human HSPC, with potential to predict efficiency as well as clinical outcome of G-CSF-induced mobilization, homing, and engraftment kinetics as well as efficiency.

Potential areas for future investigation:

- Assess the clinical importance of PAR1 by validating its role in autologous HSPC transplantation setting, where the main difficulty is harvesting mobilized HSPC from heavily chemotherapy-treated patients.
- Manipulating PAR1 expression in human HSPC to improve the efficiency of mobilization and prognosis of HSPC-transplanted patients.
- Analyze the role of coagulation proteases in G-CSF-induced mobilization.

### Kidney: The Coagulome in Kidney Disease

The loss of the microvasculature, also referred to as microvascular rarefaction, is a critical determinant in kidney disease states such as acute kidney failure, diabetic nephropathy, or kidney transplant rejection. 59 The resulting ischemia is a driver for an inflammatory response that is associated with increased expression of profibrotic mediators such as TGFβ or CTGF (connective tissue growth factor; CNN2) that ultimately contribute to chronic kidney failure. Pericytes are essential functional components of the microvasculature stabilizing the capillaries through multiple reciprocal interactions. A key mechanism in microvascular rarefaction is the dissociation of pericytes from the capillary ECs 60 subsequent to inflammatory or pro-angiogenic stimuli 61 such as tumor necrosis factor-α, vascular endothelial growth factor, or a disbalance in the circulating levels of angiopoietin(ang)-2 over ang-1. 62 Conditions associated with ischemia can rapidly upregulate TF expression by vascular EC and elicit a pro-coagulant response through activation of the endothelial PARs. As a consequence, activated ECs lose their cell–cell contacts, dissociate from the pericytes, and engage in an angiogenic response, all processes that can promote microvascular rarefaction. For instance, in AF, the disbalance between supply and the excessive need for oxygen by the fibrillating myocytes leads to a state of hypoxia 5 that promotes subendothelial TF expression. Therefore, a role for the coagulome in the microvascular rarefaction that drives the pro-fibrotic substrate for AF is under active investigation. For instance, a recent paper by Dólleman et al explored the impact of DOACs on vascular integrity in vitro using platelet-free plasma in thrombin generation and endothelial barrier assays. 63 Interestingly, they demonstrated that while the anti-FXa DOAC rivaroxaban and the antithrombin DOAC dabigatran are both efficient in blocking their target proteases, rivaroxaban could preserve endothelial barrier function while dabigatran failed to protect endothelial integrity. The barrier disrupting effect of dabigatran could be prevented in the presence of a custom-made peptide that blocks thrombin's exosite-I. The take-home message of this study is that selective use of DOACs could well have a favorable impact on long-term (micro-)vascular health.

**Fig. 1:**
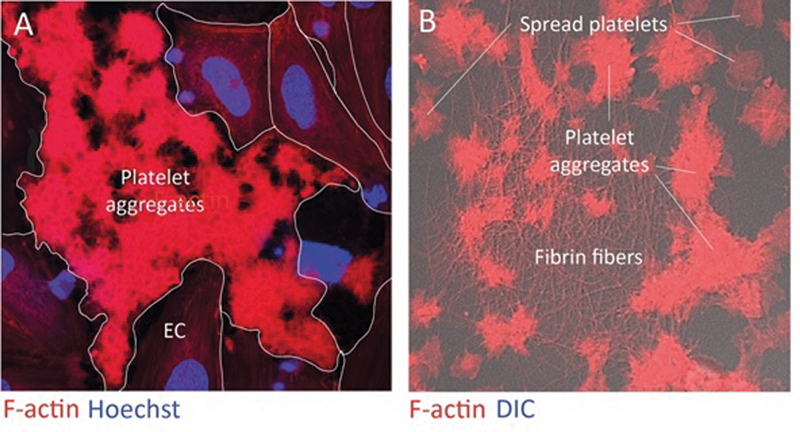
Platelet-rich plasma rotation perfusions on TNFα-treated monolayers of human umbilical vein endothelial cells. After 15 minutes the cultures were fixed and stained for platelets (F-actin), nuclei (Hoechst), and (right panel) fibrin (antifibrinogen antibody). ( **A** ) Platelets selectively adhere to the extracellular matrix exposed in gaps that appeared between the endothelial cells upon overnight exposure to TNFα. Subsequent analyses demonstrated the platelets display all characteristics of “coated platelets.” ( **B** ) Fibrin fibers confirm the activation of the coagulation system at the site of platelet adhesion. TNFα, tumor necrosis factor α.

Many studies have shown that activation of the coagulation system and platelets go hand in hand. In mouse models of kidney I/R injury, platelets rapidly adhere to the ischemic (micro-)vasculature. Using an in vitro model, it was demonstrated that platelets predominantly adhere to the (TF-rich) EC matrix where gaps were formed resulting from the loss of EC–EC contacts in cultured monolayers ( Fig. 1 ). Using this model of perfusion of platelet-rich plasma, it was demonstrated that the adhered platelets markedly stimulated the generation of FXa depending on the presence of phospholipids, TF, and TFPI (Dolleman et al, manuscript in preparation). Subsequent studies revealed that the adhered platelets resemble the so-called coated platelets 64 that, due to dual activation, highly express P-selectin, TF, TFPI, and heparinase. These data strongly support a potential role for platelets in ischemia-driven microvascular rarefaction. This could be particularly relevant for patients with diabetic nephropathy. In fact, recent data show that platelets can be detected in the glomeruli of patients with diabetic kidney disease. Moreover, a direct relation was observed between platelet-derived extracellular vesicles and the degree of albuminuria in these patients. 65 Subsequent mechanistic studies in a mouse model for diabetic nephropathy demonstrated that the platelet P2Y12 inhibitor ticagrelor could counteract disease progression by lowering albuminuria, mesangial matrix expansion, macrophage infiltration, and fibrosis. 66 Future studies with selective platelet inhibitors such as GLP-1 analogues 67 could well augment our therapeutic options in progressive ischemia-associated diseases of the kidney.

Potential areas for future investigation:

- While equally effective in anticoagulant activity, selective use of DOACs could have long-term beneficial effects for microvascular complications in chronic kidney disease patients. These in vitro findings should be validated by in vivo animal and clinical studies.
- The long-term benefit of the use of selective platelet inhibitors by patients with diabetic nephropathy warrants clinical investigation.

### Coagulation in Endothelial Cell Barrier Function

Hyperlipidemia results in LDL/APOB-containing lipoprotein accumulation in the artery walls, promoting vascular inflammation, EC dysfunction, and localized loss of endothelial barrier function. Recent works have highlighted the extensive crosstalk between coagulation and inflammation in such diseases in which EC dysfunction serves as a hallmark. 68 69 70 71 72 Yet, the inciting factors for inflammation in hyperlipidemia remain unclear. Studies have shown that inhibiting FXI reduced inflammatory markers in mouse and nonhuman primate models of either acute and chronic inflammation. 31 73 74 75 Translating this to patients, it has recently been shown that pharmacological inhibition of FXI reduces inflammatory markers, including the hallmark biomarker C-reactive protein (CRP), in a clinical trial in end-stage renal disease patients on hemodialysis. 76 Follow-on studies are underway to evaluate whether use of FXI inhibition for the prevention of catheter-associated thrombosis similarly blunts the rise in CRP levels following placement of an indwelling catheter, which would provide further evidence of a link between the FXI activation and inflammation (ClinicalTrials.gov #NCT04465760). Continuing this theme, preliminary studies in a primate model of diet-induced hyperlipidemia show that the elevated CRP levels in an obese cohort were reduced by approximately 25% following 4 weeks of anti-FXI therapy. Defining the mechanisms by which FXI plays a role in propagating inflammation will provide insight into whether FXI inhibition has potential therapeutic anti-inflammatory benefits in cardiovascular disease and, in particular, hyperlipidemia.

**Fig. 2:**
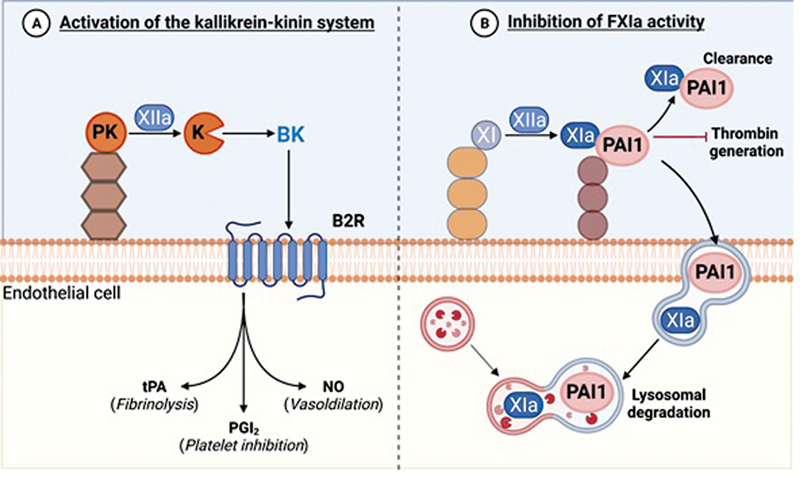
Endothelial cells promote ( **A** ) the activation of the kallikrein–kinin system while ( **B** ) inhibiting FXIa activity.

Vascular endothelium serves as a site of catalysis for enzymatic reactions, while also facilitating multiple pathways that maintain blood cells in a quiescent state. As such, EC dysfunction is common in inflammatory diseases, such as atherosclerosis, and often appears early on in the course of the disease. 77 78 Recent observations have shown that FXI inhibition preserves endothelial barrier function in mice and primates in vivo, 31 75 suggesting that the EC surface may serve as a source or a sink for FXIa activity ( Fig. 2 ). Mechanistic studies discovered that the anticoagulant role of the endothelium includes sequestration of FXIa activity. 79 Next, it was determined that FXIa is inactivated by complex formation with vascular EC-derived PAI-1. It was found that FXIa–PAI-1 complexes were either released into the media or trafficked to EC endosomes and lysosomes in vitro ( Fig. 2 ). In a nonhuman primate model of lethal systemic inflammatory response syndrome (SIRS) associated with sepsis, the authors were able to detect FXIa–PAI-1 complexes in the circulation after a bacterial challenge. 79 In preliminary studies, it was found that inactivation of FXIa by PAI-1 on the EC surface may invoke a signaling pathway to increase vascular permeability by way of cleavage of EC VE-cadherin. Taken together, these data suggest that the kallikrein–kinin system, and, in particular, FXI, act as a nexus between the coagulation cascade, inflammation, and EC barrier function. This work holds promise to provide rationale for FXI inhibition as a useful approach for protecting barrier function in settings characterized by inflammation such as hyperlipidemia.

Potential areas for future investigation:

- To determine whether the ability of FXI to act “upstream” and activate FXII contributes to activation of the kallikrein–kinin system to promote inflammation.
- To explore if FXI activation or activity directly regulates EC barrier (dys)function.

## Theme 2: Novel Mechanisms of Thrombosis

### The Relevance of Factor XII?

**Fig. 3:**
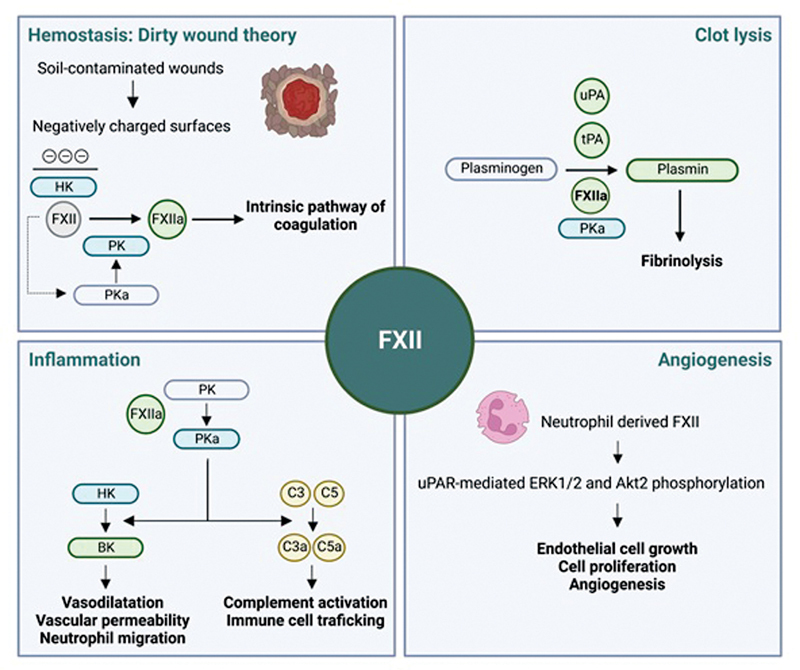
Potential physiological role of factor XII. BK, bradykinin; C3(a), (activated) complement factor 3; C5(a), (activated) complement factor 5; FXII(a), (activated) coagulation factor XII; HK, high-molecular-weight kininogen; PK, plasma prekallikrein; PKa, plasma kallikrein; tPA, tissue plasminogen activator; uPA, urokinase plasminogen activator.

FXIIa is a serine protease consisting of a heavy and a light chain held together by a disulfide bond. It auto-activates upon contact with negatively charged compounds (e.g., glass, kaolin, and diatomaceous earth), as well as biological negatively charged molecules (e.g., DNA, RNA, misfolded proteins, polyphosphates). Substrates of FXIIa include proteins involved in coagulation, inflammation, fibrinolysis, and angiogenesis. Surprisingly, however, its deficiency in humans has not been associated with an overt pathological phenotype. Nevertheless, a cohort study found FXII levels to be inversely associated with overall mortality, although not for those at the lowest levels. 80 These apparently contradictory findings have stirred the debate on the physiologic functions of FXII ( Fig. 3 ).

Involvement of FXII in human hemostasis is based on its essential role in contact-activated in vitro coagulation assays. Moreover, its concentration in blood is higher than any other coagulation factor from the contact activation system (e.g., ∼10-fold higher than FXI). This stands in stark contrast to the lack of a bleeding phenotype in FXII-deficient humans and knockout mouse models. Thus, the question arises: is FXII really a coagulation factor? To explain this discrepancy, it has been hypothesized that FXII might only be involved in hemostasis of soil-contaminated wounds, where it is activated by negatively charged silicates. This so-called “dirty wound theory” is based on the observation that marine animals lack FXII, in contrast to land-based animals. 81 From an evolutionary perspective, particularly the absence of FXII in sea mammals suggests its redundancy in wounds which are continually cleaned by surrounding water. This theory is supported by experiments in FXII-knockout mice, where hemostatic differences between clean and soil-contaminated wounds were observed. 82 Future studies will have to establish if these differences also have physiological relevance in humans.

While its hemostatic role in wound healing remains uncertain, activated FXII is known to trigger the formation of kallikrein and bradykinin release, which stimulates vasodilation, vascular permeability, neutrophil migration, and complement activation contributing to the immune defense in the wound site. Interestingly, excess FXIIa levels are observed in a genetic disease called hereditary angioedema (HAE), characterized by recurrent episodes of severe edema due to extreme bradykinin release. 83 It is caused either by a FXII mutation causing increased autoactivation or a deficiency of its main inhibitor, C1 esterase inhibitor. 84 Another mutation of FXII resulting in spontaneous auto-activation has been identified as the cause of a rare disorder termed FXII-associated cold autoinflammatory syndrome (FACAS), which is characterized by cold-induced urticaria, arthralgia, chills, headaches, and malaise. 85 These phenotypes of HAE and FACAS both support the notion that FXII is mainly involved in regulating inflammation and vascular permeability.

Notably, patients with HAE or FACAS are not reported to have increased thrombosis risk, despite the underlying uncontrolled FXII activation. This begs the question: can FXIIa “choose” to have enzymatic activity for a certain substrate? Unraveling of this question will require further molecular insight into FXII. Currently, this protein is thought about as a “string of pearls” with five domains linked to the protease domain by a proline-rich region. However, the natural confirmation of FXII is most likely very different and our understanding of individual domains is limited. Molecular research will have to establish in what ways this protein can be activated and interact with its substrates, which might explain distinct enzymatic activity in different conditions.

Furthermore, FXII has been implicated in the fibrinolytic system based on its high degree of homology with tissue plasminogen activator (tPA). Indeed, in vitro experiments have shown that FXIIa can convert plasminogen to plasmin and enhance fibrinolysis, but its rate is much lower than that of tPA or urokinase plasminogen activator (uPA). 86 Therefore, the relevance of this enzymatic activity in vivo remains to be established. Conversely, however, plasminogen was found to influence pathways of FXII presenting as HAE in the setting of a rare plasminogen mutation (HAE-PLG). 87

Finally, although FXII is mainly secreted by the liver, there is growing evidence for a separate pool of leukocyte-expressed FXII that contributes to wound healing and angiogenesis. 88 This was found to be mediated by unactivated FXII signaling through the uPA receptor, stimulating processes such as EC growth and proliferation. This more recent finding highlights the variety of roles FXII has in human physiology, some of which might still need to be uncovered.

In conclusion, although clinical data on FXII do not support a pivotal role in hemostasis or thrombosis, new perspectives regarding the role of FXII have been discovered in the last two decades. These include a role in inflammation, fibrinolysis, and angiogenesis, with novel pathways downstream of FXII still pending to be elucidated.

Potential areas for future investigation:

- To establish why gain-of-function mutations in FXII lead to an inflammatory, but not a thrombotic state.
- To further delineate the relationship between structure and function of FXII.

### Biomechanics of Fibrin and Fibrin Clot Lysis

**Fig. 4:**
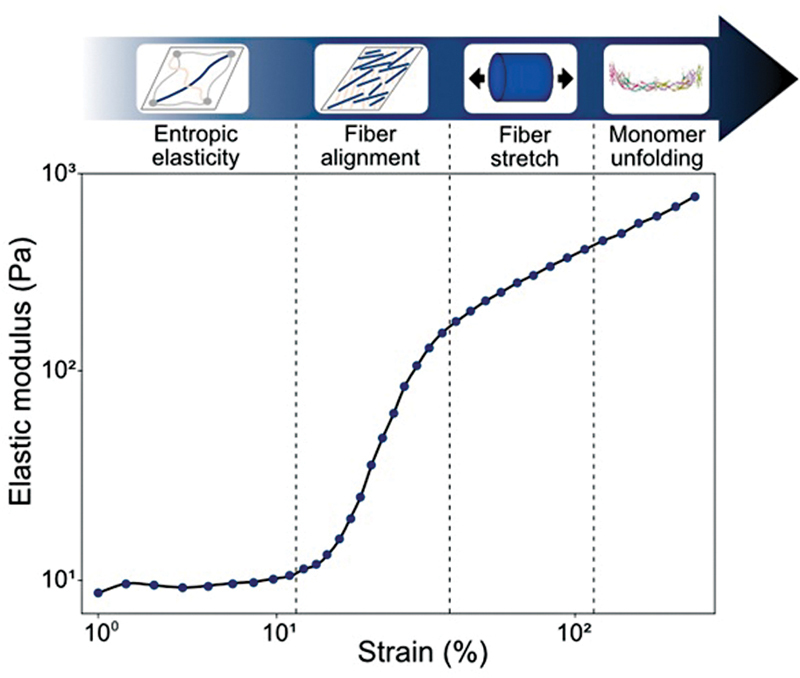
Elastic modulus of a fibrin network as a function of strain, measured by shear rheology. Fibrin forms a soft elastic network at low strain, and stiffens 100-fold in various stages marked by the vertical dashed lines when the strain is increased. The stages correspond to entropic elasticity (low strain), strain-induced fiber alignment, fiber backbone stretching, and finally fibrin monomer unfolding.

In both physiological and pathological conditions, thrombi are subjected to extreme mechanical forces such as wound stretch, clot contraction, or shear stress. Yet, thrombi manage to retain their structural integrity through a remarkable combination of compliance and resilience. These characteristics are understood to be provided by the fibrin network, which forms the primary scaffold of clots. Fibrin networks can reversibly stretch up to approximately 150%, resist elongation of several hundred percent, and stiffen by at least two orders of magnitude before rupture. 89 90 Biophysical studies over the past decade have shown that these unique mechanical features stem from the complex structure of fibrin fibers, which are bundles of protofibrils that are themselves double-stranded filaments of fibrin molecules. Consequently, fibrin networks undergo several phases of stretch at different structural levels ( Fig. 4 ). 91

At first, stretch causes the natively disordered and hence flexible αC-domains of fibrin molecules to straighten, which allows elongation (i.e., strain) with almost no increase in resistance to deformation (i.e., elastic modulus). Next, the fibers gradually align in the direction of strain, which is accompanied by a strong increase of the elastic modulus. Finally, the strain is transferred to the folded domains of the fibrin monomers, which results in a further linear increase of the modulus. If the strain continues to be increased, however, the folded monomer domains start to unfold, which provides irreversible elongation, and eventually leads to rupture.

These insights into fibrin mechanics have only recently been acquired by applying novel methods based on rheology combined with in situ X-ray scattering or vibrational spectroscopy complemented with single-fiber and single-molecule stretching assays. 90 91 92 This mechanistic understanding of fibrin mechanics is ready to be used now to assess the role of mechanical forces in thrombotic and bleeding disorders. Thus far, clot characteristics have mainly been studied using microscopy. These studies found that patients with MI, IS, VTE, and recurrent episodes are characterized by in vitro fibrin clots with a dense network and thinner fibers. 93 Such clots are known to be less permeable, making them less susceptible to fibrinolysis, which could explain associations to adverse outcomes. However, dense clots are also known to be stiffer, which potentially increases thrombus obstructiveness or embologenicity, giving an alternative explanation for differences in outcomes. In contrast, patients with hemophilia A and B were found to have clots with loose networks and thick fibers, which might make them more prone to bleeding events due to the fragility of such clots. 94 95

These findings suggest that mechanical phenotyping of clots is a promising avenue for future research. It might provide parameters that can contribute to more accurate diagnosis and risk stratification, mirroring the use of mechanical phenotyping for connective tissue disorders and cancer. 96 Also, it could give rise to novel therapies using pharmacological or mechanical interventions that influence thrombus mechanics to, for example, improve outcomes of endogenous or therapeutic thrombolysis. However, much still needs to be elucidated about clot mechanics and the role of the fibrin network in physiology and pathology. Experiments on fibrin have mostly been performed in purified systems. This means it is largely unknown how fibrin interacts with other clot components such as platelets and red blood cells, and hence how thrombus composition and spatially heterogeneous structure affect thrombus pathologies (e.g., platelet- vs. fibrin-rich thrombi). 97 98 In summary, the integration of biophysical research into the field of thrombosis and hemostasis is bound to bring fascinating fundamental insights and clinically relevant advances in the near future.

Potential areas for future investigation:

- The interplay of macromolecules including fibrin and different cell types (platelets, red blood cells) in clot mechanics and sensitivity to lysis.
- The relation between different mechanical properties of thrombi (stiffness, viscoelasticity, plasticity, rupture strength) and the risk of embolization and sensitivity of clots to lysis.
- Evaluate the potential of mechanical phenotyping of thrombi, either collected by thrombectomy or reconstituted from patient plasma, for diagnosis and risk stratification.
- Evaluate the potential of mechanical phenotyping of thrombi to assess the efficacy of novel therapies using pharmacological or mechanical interventions that influence thrombus mechanics to improve outcomes of endogenous or therapeutic thrombolysis.

### The Microbiome and Thrombosis

In contrast to acute inflammatory conditions in case of viral infections, the gut microbiota is a driver of low-grade inflammation, chronically impacting vascular inflammation. 99 Dependent on host nutrition, microbiota-derived products constantly leak into the portal circulation, with signaling-active molecules and metabolites reaching the hepatic microcirculation. 100 Studies on germ-free mouse models clearly demonstrate that the transcriptome of the liver sinusoidal endothelium is broadly influenced by gut microbial colonization, with the sphingolipid synthesis pathway recently identified as one of the primarily affected microbiota-modulated pathways. 101 102 Furthermore, vWF expression in the hepatic endothelium is augmented by the presence of gut commensals. 103 Another example is the sensitivity of neutrophils toward lipopolysaccharide (LPS)-induced neutrophil extracellular traps (NET)osis, which was attenuated by the presence of gut commensals. 104 Importantly, several experimental and clinical studies unveiled the gut microbiota as a novel risk factor for cardiovascular disease and arterial thrombosis. 103 105 106 107 Interestingly, under low-cholesterol diet conditions, germ-free *Apoe* -deficient and germ-free *Ldlr* -deficient mice had elevated plasma cholesterol levels and *Apoe* -deficient mice presented increased atherosclerotic lesion size, an effect that was abolished at high-cholesterol diet feeding. 108 109 110 Interestingly, in the germ-free *Apoe* -deficient mouse atherosclerosis model, *Roseburia intestinalis* , due to its production of the short-chain fatty acid butyrate, has a protective role in atherogenesis. 111 Another microbiota-derived metabolite related to cardiovascular risk and arterial thrombosis is trimethylamine (TMA), a choline metabolite produced by TMA-lyase enzymes and converted to trimethylamine-N-oxide (TMAO) by flavin-dependent monooxygenase-3 in the liver. 112 113 114 The metaorganismal TMAO-pathway was demonstrated to promote arterial thrombus growth via multiple pathways, including the induction of platelet hyperreactivity and vascular endothelial TF expression. 106 115 Of note, in a translational pig model it was recently demonstrated that the reduction of dietary fat for a time period of 30 days, resulting in reduced plasma cholesterol levels, was able to revert dysbiosis of the fecal microbiome and to reduce plasma TMAO levels, 116 a predictive functional marker for adverse cardiac events. 117 Vascular innate immune signaling, triggered by microbial-associated molecular patterns derived from the intestinal microbiota, for instance by the activation of endothelial Toll-like receptor-2 signaling in the liver resulting in enhanced vWF synthesis, is an additional mechanism linking the gut microbiota with enhanced arterial thrombus growth. 5 In contrast to germ-free mice, colonized mice showed increased ADP-induced GPIIb/IIIa activation and elevated adhesion-dependent phosphatidylserine exposure, promoting arterial thrombus growth. 102 118 Intriguingly, gut microbial diversity might even affect cardiovascular disease therapies as shown for ticagrelor by a recent study on the efficacy of antiplatelet treatment in STEMI. 113 Moreover, it was shown that chronic statin therapy is linked to lower prevalence of microbiota dysbiosis. 119 120 In addition to above, abnormal gut microbiome homeostasis could be linked to development of chronic effects from viral infections. 121 Alterations in gut microbiome have been reported, linked to cytokine release from cells, due to viral load, with implications also seen in SARS-CoV-2 (severe acute respiratory syndrome coronavirus 2) infections. 122 123 Also, circulating extracellular vesicles potentially transport viral miRNA in the gut, further promoting dysbiosis. 124 Extracellular vesicles, carrying cytokines and pro-inflammatory markers, may also further exacerbate atherosclerosis and viral infections, such as during coronavirus disease 2019 (COVID-19). 125 126

Potential areas for future investigation:

- Based on gnotobiotic experimentation and insights from sequencing and multi-omics studies, it will be interesting to reveal microbiota-triggered molecular and cellular mechanisms involved in thrombogenesis at various settings.
- Given the broad interference of microbiota-derived metabolites with host metabolism and the microbiota-dependent regulation of host metabolic pathways involved in cardiovascular disease development, an improved understanding of their role in cardiovascular disease and thrombosis is needed.
- Well-designed functional studies are needed to identify microbiota–drug interactions, which, dependent on microbiome composition, can influence the outcome of antithrombotic therapies.

### Viruses and Coagulation: The Case of COVID-19

Viral infections are associated with coagulation disorders, driven by inflammatory pathways. 127 128 All aspects of the coagulation cascade, primary hemostasis, coagulation, and fibrinolysis, can be affected and the net result may be bleeding 129 and/or [athero]thrombosis. 130 The spectrum of viral infections comprises different dynamics, ranging from acute to chronic and from a mild to a severe clinical course, resulting in a different interplay between the inflammatory and coagulation cascades and with different risk profiles for thrombo-embolic and/or bleeding complications. The interaction between infection, inflammation, and the hemostatic system is a multifactorial dynamic process led by modifiable and nonmodifiable risk factors. Unlike most bacterial infections treatable with specific antibiotics, no specific antiviral treatment is available for most viral infections, other than supportive treatment. Otherwise, the success of treatment interventions such as dexamethasone or anti-IL-6, depends much on timing and it is a challenge to define the optimal moment or time period of intervention in a heterogeneous patient population. Investigation of coagulation disorders related to different viral infections has not been performed uniformly; therefore, common pathways are not fully elucidated yet. Furthermore, research is hampered due to specific biosafety facilities needed to study specific viruses. A better insight into pathogenesis on the one hand and improvement of bedside monitoring tools on the other hand is urgently needed to improve clinical management.

An increasing body of evidence demonstrates extensive and bidirectional interactions between inflammation and coagulation. 127 131 132 133 134 135 136 Normally, coagulation is balanced by pro- and (natural) anticoagulant mechanisms. Inflammation impacts the initiation, propagation, and inhibitory phases of blood coagulation. 132 In viral and bacterial infections, this can actually lead to both thrombotic and hemorrhagic complications. Pathogens, as well as inflammatory cells and mediators, can induce the expression of TF on monocytes and EC surfaces. 136 Direct or indirect activation of the endothelium by viruses or other pathogens may result in alterations in the coagulation and fibrinolytic systems. 137 138 There is also an incompletely understood connection of infections with RNA viruses activating toll like receptor (TLR) 7 and autoimmune antibody production. 139 These antiphospholipid autoimmune antibodies also develop in severe COVID-19 disease. 140

The clinical picture of altered coagulation in several viral infections manifests itself in bleeding (hemorrhage), thrombosis, or both. An exaggerated response may even lead to disseminated intravascular coagulation (DIC) with the formation of microvascular thrombi in various organs. 141 DIC contributes to multiple organ failure and is associated with high mortality in both bacterial and nonbacterial diseases. 134 141 It is not yet clear why some viruses cause hemorrhaging (e.g., Ebola), while others are associated with thrombosis (e.g., cytomegalovirus) and yet others show both complications (e.g., varicella zoster virus). 142 143 144 Bleeding may be aggravated by the occurrence of thrombocytopenia either separately, or as part of viral coagulopathy. 128 In addition to this, the bleeding complications of hemorrhagic viruses vary in severity, such as the minor bleeding complications in some forms of dengue and more severe bleeding in Ebola and Marburg. As mentioned for many viral infections, targeted therapy is not available, and only supportive care can be provided. In many mild cases, treatment may not even be necessary. However, to improve therapy and supportive care for complicated viral infections, a better understanding of the pathogenesis of bleeding and thrombotic complications due to viral infections is needed.

### The Case of COVID-19

In patients with severe COVID-19 infection, many studies have shown that not the infection itself, but the host immune response results in a hyperinflammatory state, which can be a trigger of vascular thrombotic events, a phenomenon that we call immunothrombosis. 145

The term thromboinflammation is derived from thrombosis associated with inflammation and is used to describe pathophysiologic perturbations due to vascular endothelial injury and/or loss of antithrombotic and anti-inflammatory functions. 146 Both cellular and humoral inflammatory mechanisms of immune surveillance are activated in this dynamic process. In acute infections, thromboinflammation may culminate in microvascular thrombosis, which is the hallmark of the disease, as has been reported in postmortem studies of patients with acute respiratory distress syndrome due to pathogens invading the respiratory tract and provoking an inflammatory response associated with acute lung injury. 147

Immunothrombosis, if balanced, is a physiological role in host defense. The term describes the microvascular thrombotic response that facilitates microbe containment and elimination, a critical component of innate immunity. 148 149 The pathological entity from immunothrombosis is in situ pulmonary thrombosis which is a different entity from the embolic events from deep vein thrombi which are a net result of thromboinflammation. 150 As part of any inflammatory response to attenuate microbial invasion, microcirculatory thrombosis also produces multiorgan injury. 151 152 These important host defense mechanisms have been described, but with the ongoing pandemic and massive numbers of COVID-19 patients who manifested lung or multiorgan dysfunction, the concept of immunothrombosis was increasingly reported. 148 In summary, although thromboinflammation and immunothrombosis have many similarities, they should not be used as interchangeable counterparts, even if they have been used synonymously in the past.

Long COVID defined as long-lasting multiorgan symptoms that last for weeks to months after SARS-CoV-2 infection is associated with cardiovascular manifestations including peri-myocarditis. If and how in situ thrombosis does play a role in long COVID is still unanswered, and studies are ongoing. Currently there is no guided therapy for long COVID other than anecdotal reports and further studies are needed to unravel the underlying mechanisms. 153

Potential areas for future investigation:

- Determine the viral or inflammatory triggers for either thrombosis and/or bleeding.
- The role of vascular bed-specific hemostasis in viral infections.
- Study the role of inflammatory components, i.e., virus-specific T-cells in the initiation and regulation of the hemostatic balance.
- Determine better ways of translating results from the homogeneous [experimental] models into clinical practice, or heterogeneous reality to improve the timing and type of therapeutic interventions.

## Theme 3: How to Limit Bleeding Risks: Insights from Translational Studies

### Genetics and Bleeding Disorders

Hemostasis is controlled by interplays between platelets, coagulation, and fibrinolysis; their normal function is to prevent bleeding. Genetic variants in genes that encode for regulators of these three processes are known to cause inherited forms of bleeding. The summary deals with the use of next-generation sequencing (NGS) approaches for diagnostic and gene discovery. To date, almost 100 curated disease-causing genes have been identified to cause inherited bleeding, platelet, or thrombotic disorders ( www.isth.org/page/GinTh_GeneLists). ^154^ This is a dynamic list that is yearly updated as since 2011; 25 novel genes have been discovered using NGS approaches. 155 This gene list is useful for clinical laboratories that have implemented multigene panel tests to diagnose inherited bleeding disorders. This is a cost-effective approach to rapidly screen patients. The international study ThromboGenomics has shown that the diagnostic rates obtained for thrombocytopenia, platelet function, and coagulation disorders are 47.8, 26.1, and 63.6%, respectively, while this rate drops to 3.1% for patients with unexplained bleeding disorders (having normal laboratory test parameters) using a multigene panel test. 156 These differences can be explained by the inclusion criteria and the quality of the laboratory test that detects the abnormality. Patients with abnormal test data for (anti-)coagulation parameters or with low platelet counts are easy to identify, and genetic variants are often associated with such defects. Still, genetic variants were also detected in some patients with normal laboratory parameters where these assays were unable to detect the defect. In contrast, the genes for the platelet function disorder “storage pool disease” or having unexplained bleeding disorder are still unknown and therefore, screening with a multigene panel test is not useful as exemplified by causing a very low diagnostic rate in the ThromboGenomics study. Of interest is the unexpected finding of oligogenic inheritance where patients have more variants in more than one gene. Today, this field still struggles with the detection of numerous variants of unknown significance (VUSs) that cannot be used in clinical practice. 157 These VUSs require further functional and genetic studies to prove pathogenicity. Rapid screening models and data exchange with the community could improve the variant classification.

International studies BRIDGE-BPD and NIHR BioResource have used whole-exome sequencing (WES) and whole-genome sequencing (WGS) for the discovery of novel genes for bleeding disorders. 158 159 Success rates are typically high if screening consanguineous or very large pedigrees, or if more families have been recruited with similar gene phenotypes. Even if the genetic defect is discovered, it can take several years to understand the disease as illustrated for SRC-related thrombocytopenia. 154 Five years after the discovery of the SRC gain-of-function variant E527K, the same variant was detected in other pedigrees that helped to delineate the syndromic phenotype associated with thrombocytopenia and RNAseq provided evidence for defective interferon regulation as underlying cause. 154 Still many patients do not receive a diagnosis even though their complete genome has been analyzed. This can be explained by the fact that each genome contains numerous unique coding variants and the noncoding regions are very difficult to analyze due to the lack of information about regions of interest (promoter or regulatory regions) versus junk DNA. An additional layer of information will be critical to understand noncoding variation. Therefore, blood cell RNAseq will be performed for patients who do not have a diagnosis but from whom WGS data are available. Gene expression and splicing analysis will assist in the understanding of variants that influence these processes as the cause of a bleeding disorder.

Potential areas for future investigation:

- Oligogenic inheritance is unexplored in our field. It is currently not understood what the clinical relevance is of combining common and rare variants in different known genes that modify bleeding and thrombosis risks. This might be relevant for molecular diagnostics as it is known that single variants can result in a different clinical severity of a certain disorder.
- Some patients present with obvious clinical bleeding phenotypes but have normal laboratory test data. Genetic causes still remain unknown for such patients as it is very difficult to find causative genes if no idea of the underlying defective pathway is known. It might be necessary to develop better laboratory assays to study such patients and these should include ECs that are currently not studied.
- In addition to the currently used WES and WGS, other OMICS methods will be required to explore disease mechanisms and enhance gene discoveries. Novel statistical methods that can combine OMICS results will be required to address these needs.

### Genetics and Antithrombotics: Toward Individual Drug Tailoring

#### Personalized Antithrombotic Therapy Based on Genetic Testing

Besides several other factors (i.e., body weight, diabetes, etc.) genetic polymorphisms play a role in the variable response of drugs in patients. 160 Therefore, genetic testing may influence the efficacy and/or safety of antithrombotic treatment, and thus optimize patients' outcomes.

#### Genetic Testing

When a nucleotide change in a gene is present in more than 1% of a population, it is called a genetic polymorphism. These polymorphisms often affect the drug-metabolizing cytochrome P450 (CYP450) enzymes, which play a role in activation or deactivation of a drug. 161

In patients with ACSs or undergoing percutaneous coronary intervention (PCI), DAPT with aspirin (acetylsalicylic acid) and a P2Y12 inhibitor (ticagrelor, prasugrel, and clopidogrel) is the cornerstone of medical therapy to prevent the recurrence of thrombotic events including stent thrombosis. 162 Ticagrelor and prasugrel are much stronger than clopidogrel and have shown reduced thrombotic events in large outcome trials. 163 164 However, the reduced thrombotic risk is counterbalanced by an increased bleeding risk. 163 164 In addition, it is well known that major bleeding has a similar impact on patient outcome as a recurrent thrombotic event, e.g., MI. 165 166 Aspirin is metabolized by different enzymes, but up to now none of the genetic polymorphisms has impacted clinical outcome. 167 Ticagrelor is a direct-acting drug, while both clopidogrel and prasugrel need activation by cytochrome CYPP450 genes. 168 The active compound of ticagrelor is metabolized by CYP3A4, which can also directly bind to the P2Y12 receptor. 169 Prasugrel is metabolized mainly by CYP3A4 and CYP2B6, and to some extent by CYP2C9 and CYP2C19; however, polymorphisms in these genes are not related with a heightened thrombotic risk. 170 However, clopidogrel is very much affected by polymorphisms which lead to less response in 30% of patients. 171

**Fig. 5:**
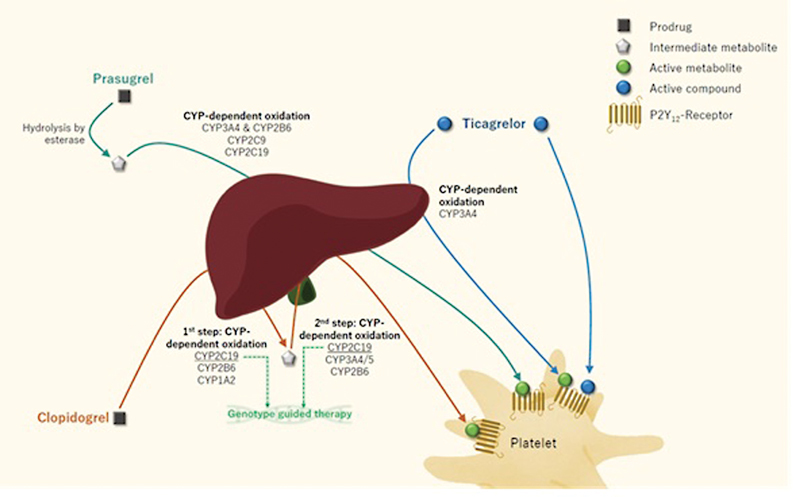
Biotransformation and metabolization of the oral P2Y12-inhibitors. Antithrombotic therapy can be personalized by (1) using CYP2C19 genotype-guided therapy, which is the only genetic polymorphism for which a genotype-guided therapy is assessed in randomized clinical trials or (2) assessing the actual responsiveness to antiplatelet therapy by measuring on-treatment platelet reactivity, which is influenced by different modifiable and nonmodifiable factors.

In the two-step activation of clopidogrel process, multiple CYP enzymes play a role (CYP2C19, CYP3A4/5, CYP1A2, CYP2B6, CYP2C9) ( Fig. 5 ). 172

CYP2C19 plays a role in both steps and is the greatest contributor in this metabolic process. The prevalence of the CYP2C19 polymorphisms (*2 and *3) is approximately 25% of the Caucasian population. 168 There are much data demonstrating that carriers of CYP2C19 LoF-alleles have a diminished antiplatelet response and therefore higher platelet reactivity (HPR). 173 174 175 176 This HPR translates to higher risk for thrombotic events, including stent thrombosis. 173 177

#### Clinical Evidence for a Genotype-Guided Antithrombotic Therapy

Many studies have assessed a CYP2C19 genotype-guided strategy (escalating or de-escalating) in patients with coronary artery disease (CAD). 178 179 De-escalation means switching from a more potent drug (ticagrelor or prasugrel) to the less potent clopidogrel in extensive metabolizers, while escalation means switching from clopidogrel to ticagrelor or prasugrel in intermediate or poor metabolizers. De-escalation can be used in ACS, where standard treatment is ticagrelor. Escalation can be done in chronic coronary syndrome patients undergoing PCI, stroke or peripheral artery disease, where clopidogrel is standard treatment.

In the RCT the Popular Genetics, a genotype-guided de-escalation strategy was tested versus usual care in 2,488 patients undergoing primary PCI for STEMI. All patients were treated with aspirin, but in the genotype-guided group, intermediate and poor metabolizers were treated with ticagrelor or prasugrel (39%), and extensive metabolizers with clopidogrel (61%). Patients in the control group were all treated with ticagrelor or prasugrel. Genotype-guided P2Y12-inhibitor treatment reduced the bleeding risk (9.8 vs. 12.5%, hazard ratio [HR]: 0.78, 95% confidence interval [CI]: 0.61–0.98, *p* = 0.04) and there was no increase in thrombotic events.

In the RCT TAILOR-PCI, 5,302 patients undergoing PCI for ACS or stable CAD were randomized to genotype-guided escalation or conventional therapy (clopidogrel). 180 In the genotype-guided group, intermediate or poor metabolizers were treated with ticagrelor (31%), and the other patients were treated with clopidogrel (68%). The primary analysis was only in patients who were intermediate or poor metabolizers and did not show a statistical difference in cardiovascular death, MI, stroke, stent thrombosis, and severe recurrent ischemia at 12 months (HR: 0.66, 95% CI: 0.43–1.02; *p* = 0.06), but the reduced event rate suggests a clinical benefit with the genotype-guided group. There was also no significant difference in bleeding between groups. Despite the fact that the trial was underpowered to detect an effect size less than the prespecified expected 50% relative risk reduction, it showed a promising reduction in thrombotic risk of genotype-guided therapy. A meta-analysis including 15,949 patients with CAD showed that in intermediate or poor metabolizers, ticagrelor/prasugrel reduced thrombotic risk as compared with clopidogrel, but in extensive metabolizers there was no difference in thrombotic risk whether patients were treated with ticagrelor/prasugrel or clopidogrel. 178 179 Therefore, the Clinical Pharmacogenetics Implementation Consortium recommends to avoid clopidogrel in intermediate and poor metabolizers and use prasugrel or ticagrelor 178 ( Table 1 ). Nevertheless, genotype-guided antiplatelet therapy is not yet standard care in patients with CAD, although genotype-guided de-escalation of P2Y12 inhibition has a class IIb guideline recommendation and can be considered for ACS patients deemed unsuitable for potent platelet inhibition, i.e., with a high bleeding risk. 181

**Table 1:** Overview of the different CYP2C19 phenotypes with the coherent CYP2C19 diplotypes and the antiplatelet therapy recommendations when considering clopidogrel for cardiovascular indications

Phenotype	CYP2C19 diplotypes	Response to clopidogrel	Therapeutic recommendation
Ultra-rapid metabolizer (UM)	*17/*17	Normal or increased antiplatelet response to clopidogrel	If considering clopidogrel, use at standard dose
Rapid metabolizer (RM)	*1/*17	Normal or increased antiplatelet response to clopidogrel	If considering clopidogrel, use at standard dose
Extensive metabolizer (EM)	*1/*1	Normal antiplatelet response to clopidogrel	If considering clopidogrel, use at standard dose
Intermediate metabolizer (IM)	*1/*2, *1/*3, *2/*17 or *3/*17	Reduced antiplatelet response to clopidogrel	Avoid standard-dose clopidogrel. Use prasugrel or ticagrelor at standard dose if no contraindication
Poor metabolizer (PM)	*2/*2, *2/*3 or *3/*3	Significantly reduced antiplatelet response to clopidogrel	Avoid clopidogrel. Use prasugrel or ticagrelor at standard dose if no contraindication

Based on the above-presented evidence, some centers have implemented a genotype-guided strategy for P2Y12 inhibition. 182 Their results are in line with previous meta-analyses and thus promising.

Most evidence for genotype-guided antiplatelet treatment was obtained in patients with CAD. Nevertheless, other vascular patients sharing the same pathophysiology may also benefit from genotyping. A meta-analysis in patients with IS or TIA demonstrated that intermediate and poor metabolizers of clopidogrel have a higher risk of recurrent stroke. 183 These results are supported by the RCT CHANCE-2, demonstrating in 6,412 patients with acute IS or TIA, who were intermediate or poor metabolizers of clopidogrel, that ticagrelor reduced thrombotic risk as compared with clopidogrel. 184

### Clinical Rationale for Antagonizing Antithrombotic Agents in Bleeding Patients

#### Novel Reversal Agents

Although the DOACs have considerably improved anticoagulant treatment, the risk of bleeding is still present. Importantly, all bleeds are multifactorial in nature depending on an interaction of modifiable and nonmodifiable risk factors. 185 186 Furthermore, ethnic differences may play a role, as recently discussed for Asian populations and antithrombotic medication. 187 This implies that the presence of an anticoagulant, whether a vitamin K antagonist (VKA) or a DOAC, is merely a contributing factor, rather than a causal one.

Rapid reversal of the anticoagulant effect of DOACs may therefore be required in the case of life-threatening bleeding, emergency surgery, or severe trauma. Prothrombin complex concentrates (PCCs) and recombinant FVIIa (rFVIIa) have the ability to overcome the anticoagulant effects of DOACs. More recently, specific reversal agents have been developed that act as a decoy and scavenge the thrombin and FXa inhibitors. Idarucizumab is a monoclonal antibody fragment that tightly binds to and effectively counteracts the anticoagulant action of dabigatran. 188 For the FXa inhibitors, andexanet alfa was developed, a modified FXa molecule that lacks the phospholipid-binding Gla domain, and has its active site mutated to prevent enzymatic activity. 189 Both idarucizumab and andexanet alfa have been registered, although not everywhere in the world. Since both idarucizumab and andexanet alfa have to bind to their target, they have to be in excess of the circulating anticoagulant and consequently large quantities have to be administered, which is one of the reasons that their use is associated with high costs. Also, these agents are specific for their target and knowledge about DOAC intake has to be available before reversal can be initiated. The search for novel reversal agents for anticoagulant drugs is therefore continuing.

Table 2 summarizes the available reversal agents and several novel reversal agents that are currently under development. Scavenging proteins such as gamma-thrombin-S195A (for dabigatran or potentially other antithrombin anticoagulants) 190 and Gla-domainless FXa-α2-macroglobulin (for anti-FXa anticoagulants), 191 interact with the small-molecule anticoagulants and have been shown to be effective in vitro and in animal models. Alternatively, several hemostasis-enhancing proteins have been identified, characterized, and tested in vitro and in vivo. Examples for this approach are modified FX(a) molecules, such as FXa-I16L, FX-C and FX-Phe174, and so-called superFVa.

**Table 2:** Overview of reversal agents. The agents are categorized in reversal of dabigatran (anti-IIa), anti-Xa anticoagulants or with universal action. Furthermore, the agents were divided in proteins or small molecules and by mechanism of action (decoy or non-decoy). (  ): protein-based, decoy; (  ): protein-based, non-decoy; (  ): small molecule, decoy; (  ): small molecule, non-decoy.

Anti-IIa	Anti-Xa	Haemostasis enhancing	Universal
Idarucizumab ( )	Andexanet alfa ( )	FVIIa ( )	Ciraparantag ( )
Gamma-thrombin-S195A ( )	GladomainlessFXa-alfa2M ( )	(a)PCC ( )	OKL-1111 ( )
		FXa-I16L ( )	
		FX-C ( )	
		FX-Phe174 ( )	
		SuperVa ( )	

FXa-I16L is a FXa molecule that is zymogen-like and therefore resistant to active-site inhibitors. 192 Its activity is restored after binding to FVa and is thereby more potent than decoy FX molecules. Because of its potent hemostatic-enhancing effect, it not only counteracts FXa inhibitors, but also thrombin inhibitors. This variant has been tested in a phase 1 clinical trial, appeared safe and well-tolerated, 193 and demonstrated a dose-dependent procoagulant effect.

FX-C is a chimera of human FX with an inserted 99 loop of snake FX from *Pseudonaja textilis* . 194 This makes the molecule insensitive to FXa DOACs. Functionality has been proven in vitro and in vivo, and the molecule is currently undergoing phase 1 testing (source: VarmX Web site).

SuperFVa is an aPC-resistant FVa variant with three mutations: Arg306/506/679Gln. 195 In addition, a disulfide bond has been inserted between the A2 and A3 domains to enhance stability. SuperFVa improved thrombin generation in plasma and reversed bleeding by both FXa and thrombin inhibitors in mice. 196

Apart from protein approaches, there are currently two small molecules in development as reversal agents. Ciraparantag, a small molecule that specifically binds to the DOACs and heparin, acts rapidly and reduces bleeding induced by these anticoagulants in animals. 197 In humans, it is well tolerated. 198 Major disadvantage of the (clinical) use of ciraparantag is that it can only be monitored with a whole blood clotting time, since it binds to citrate in collection tubes and to clotting reagents that are normally used in the coagulation laboratories.

Another small molecule under development is OKL-1111. This is a cyclodextrin that does not initiate coagulation, but enhances thrombin formation in both the absence and presence of anticoagulants. In bleeding models in animals, it could be demonstrated that reversal was obtained toward DOACs, low-molecular-weight heparin, VKAs and clopidogrel (Meijers, unpublished observations) making it a truly universal reversal agent. Phase 1 studies are planned for 2023.

Potential areas for future investigation:

- Determine which of the characteristics of the novel reversal agents (specific or universal, small molecule or protein, decoy or nondecoy) will be leading in the choice for the best reversal agent.
- The next hurdle will be the demonstration of improved clinical outcome of novel reversal agents compared with PCC, idarucizumab, or andexanet alfa in patients presenting with serious bleeding or requiring urgent intervention or surgery.

## Theme 4: Hemostasis in Extracorporeal Systems: The Value and Limitations of In Vitro Models

### Assessing Thrombosis and Hemostasis Ex Vivo

Evaluation of the hemostatic process in preclinical as well as clinical settings becomes increasingly important in the assessment of the thrombotic or bleeding risk in patients. The routine hemostasis assays in the clinical diagnostic laboratory are imperative for the screening and diagnosis of hemostatic abnormalities and for monitoring the effectiveness of antithrombotic therapies, especially in high-risk patients. Although most widely used point-of-care assays like whole blood aggregometry and coagulation tests (prothrombin time, activated partial thromboplastin time [aPTT]) can detect severe hemostatic defects and effects of pro- and antithrombotic drugs, these assays lack sensitivity and fail to measure the interdependency of hemostatic pathways, i.e., platelet activation, coagulation, fibrin formation, and fibrinolysis, in clot formation 199 200 201 ( Table 3 ). In an effort to include as many components of the hemostatic system as possible, more robust and global assays were developed such as thrombin generation assays, viscoelastic assays (thromboelastography/-metry), and microfluidic models. 199 202 203 Some of the global assays, like thrombin generation and thromboelastometry, have demonstrated potential to improve the identification of patients on antithrombotic drugs who are at risk of bleeding. 204 205 Still, clinical applicability of these global assays is difficult due to (pre-)analytical variables, duration of test procedure, and interpretation of test results.

**Table 3:** Overview of hemostatic parameters and the corresponding clinical tests

Hemostatic factor/process	Corresponding test
Platelet adhesion	Platelet function analyzer (PFA)
Platelet secretion	Lumiaggregometry (ATP release) Flow cytometry (P-selectin)
Platelet aggregation	Aggregometry (e.g., light transmission aggregometry, multiple electrode impedance aggregometry) Platelet function analyzer (PFA) Flow cytometry
Coagulation	PT, aPTT, thrombin generation Viscoelastic methods (e.g., ROTEM, TEG) Coagulation factor determination
vWF	Platelet function analyzer (PFA) Platelet agglutination assay vWF antigen and activity assay
Hematocrit	Hematology analyzer
Shear-dependent platelet function	Platelet function analyzer (PFA)
Vasoconstriction	No test available, bleeding time is obsolete

Abbreviation: aPTT, activated partial thromboplastin time; PT, prothrombin time; ROTEM, rotational thromboelastography; TEG, thromboelastography; vWF, von Willebrand factor.

**Fig. 6:**
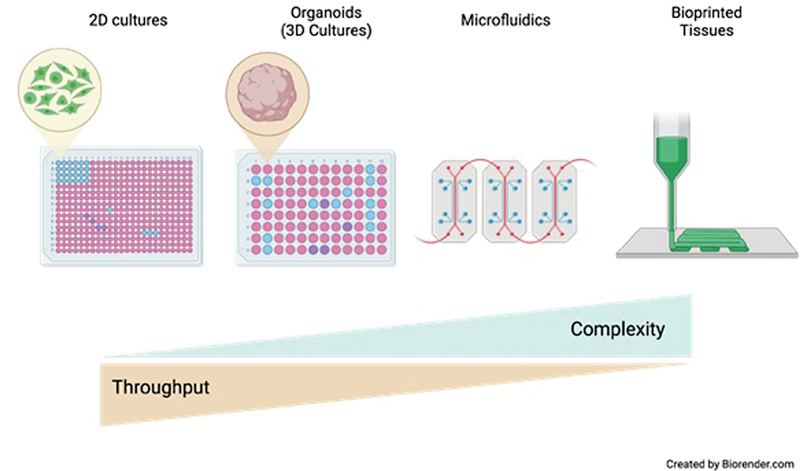
Balancing system complexity with throughput to meaningfully address biological and translational questions. With the development of progressively more physiologically relevant and complex in vitro models, there is a concurrent decrease in throughput which has significant implications for addressing biological questions. A key consideration will be maintaining scalability, both experimentally and in terms of cost, as improved 3D cultures, microfluidic platforms, and bioprinted models are developed.

Microfluidic flow devices have been used for decades mainly in research, enabling the simultaneous assessment of platelet and coagulation activation under flow conditions. 206 In addition, endothelialized models allow to study effects of endothelial barrier function and endothelial activation on hemostatic processes, providing a more physiological approach to assess the risk of bleeding or thrombosis ex vivo ( Fig. 6 ). 207 208 This has improved patient diagnostics and our understanding of inherited or acquired hemostatic abnormalities tremendously. However, standardization of such assays and their (routine) use in clinical diagnostics remains challenging, in spite of previous efforts from international scientific committees and the general consensus on the need for standardization. 209 Reasons for the lack of standardization include the complicated and time-consuming (pre) analytical handling of the (endothelialized) assays, alongside with high costs currently associated with available assays. Important aspects to enable translation of flow-based assays into clinical diagnostics or treatment monitoring include:

- The full automation in (pre)analytical handling.
- Easy-to-use software applications (development using artificial intelligence-based algorithms, integration of bioinformatics).
- Fast, user-independent output.
- Cost-effectiveness.
- Manufacturability and implementation of quality control measures.
- Clinical validation of microfluidic assays.

### Multicenter Studies—Committees

Applying global assays in multicenter studies will reveal the clinical value and applicability of (a combination of) these assays for risk prediction, diagnosis, and treatment. These multicenter studies could accelerate the standardization of novel flow-based tests by providing access to large datasets and thereby allowing assessment of test variation between centers. In addition to achieving standardization at the level of manufacturing, sample preparation, data extraction, and analysis, such studies can define patient populations that benefit from novel assays. In line with these goals, large multicenter studies come with swift recruitment of the appropriate patient population and adequate power. Thereby, setting of reference ranges can be established relatively easy for the general population and specific disease states. When implementing global assays, evaluation of the net clinical benefit will be an important aspect to support the coverage of health care costs by health insurance companies. Moreover, funding for such efforts could be provided and/or supported by pharmaceutical companies, as the developed and tested global assays can also be used to test potential novel antithrombotic, pro-hemostatic drugs and antidotes in earlier phases. Approval processes of novel tests come with inherent challenges, but these can be tackled by involving expert committees that participate in clarifying and streamlining the process. Therefore, international scientific committees can initiate and oversee studies and publish results in standardized, internally validated ways (e.g., Scientific and Standardization Committee of International Society of Thrombosis and Hemostasis) along with consensus statements so that petitioners for approval can follow a more efficient process.

Potential areas for future investigation:

- To further develop global hemostasis assays that encompass all aspects of hemostasis and to bring these from a research setting toward a clinical setting.
- To standardize global hemostasis assays and their corresponding analyses for the screening and diagnosis of hemostatic abnormalities.
- To define the optimal combinations of global and routine hemostasis tests for specific clinical questions or settings.

### Extracorporeal Circuits and Hemostatic Challenges

Extracorporeal membrane oxygenation (ECMO) is a form of temporary life support for patients with severe but potentially reversible lung and/or heart failure, unresponsive to optimal conventional care. The ECMO machine provides blood oxygenation (veno-venous, V-V ECMO) or both oxygenation and circulatory support (veno-arterial, V-A ECMO) with an artificial circuit and membrane, thereby taking over the circulatory and respiratory functions. 210 Thus, ECMO secures support while the health care team works on treating the underlying disease or until organs for transplant become available.

Although ECMO represents a potentially lifesaving therapy and its increase in clinical practice has mirrored a rapid expansion of research on this technology, it still retains intrinsic side effects and complications due to the artificial materials required and its effects on the circulatory, endothelial, hematologic, inflammatory, and immune systems.

Complications in patients receiving ECMO therapy are common and can be associated with worse outcomes. 211 In particular, current rates of bleeding events are unacceptably high and reported to occur in approximately 30% of patients, 212 with a 10% risk of major bleeding and 4 to 10% risk of intracranial hemorrhage. 213 214 Bleeding events independently impact patient prognosis, including mortality. 215 216 217

Patients undergoing ECMO support are predisposed to bleeding through various mechanisms, and these can be classified into patient, treatments, and circuit-related. Many factors that may place patients undergoing ECMO at higher risk of bleeding have been identified, 218 including underlying critical conditions prompting ECMO initiation, comorbidities, multiorgan dysfunction, and the technology itself. 218 219 220 The contact between the patient's blood with the ECMO circuit and the SIRS lead to activation of the coagulation cascade, with effects on fibrinolysis, thrombin formation, and platelet function. 218 221 222 These changes to hemostatic balance result in the coexistence of both thrombotic and hemorrhagic risks, and the final effects may be difficult to predict. Moreover, although anticoagulation remains a standard practice in patients undergoing ECMO, 223 224 thrombotic events have been identified in approximately 15% of ECMO courses 225 and might complicate ECMO therapy with significant morbidity and mortality.

Despite the increasing clinical experience and research data available, much is still unknown about best practices and risk minimization in patients receiving ECMO therapy. In addition, our current knowledge and understanding of what predisposes patients on extracorporeal circuits to bleeding or thrombosis are poor. Therefore, advancement in prevention and early recognition of hemostatic complications, both hemorrhage and thrombosis, is essential to improve the management and outcomes of patients undergoing ECMO. A genetic predisposition to coagulation disorders in these settings, where blood and body are exposed to artificial surfaces, is already well-known but still poorly investigated and might represent an additional target for future research.

Unfortunately, there is a lack of consensus regarding the most suitable approach to best identify risk factors, especially in very sick patients, and genetic screening, while attractive, may not be proven fruitful.

Truth be told, since both ECMO patients and ECMO technology imply the involvement of multiple variables and biological pathways, our current clinical practice may suffer from a compartmentalized approach. Therefore, cooperation between basic scientists and clinicians is very much needed to bridge the gap, tackle the challenges, and reply to the compelling questions that are still waiting to be answered. While historically the well-known strategy “divide et impera” has been used by empires to succeed in expanding their territories, the scientific community should come together and share our knowledge and resources to thrive.

Potential areas for future investigation:

- Current rates of bleeding in patients treated on extracorporeal circuits are unacceptably high.
- Our current knowledge and understanding of what predisposes patients on extracorporeal circuits to bleeding or thrombosis are poor.
- Cooperation between basic scientists and clinicians is needed to bridge the gap to enable the difficult questions that need to be answered regarding the use of extracorporeal circuits.
- Lack of consensus on prioritizing those studies that would best identify risk factors, especially in patients who are very sick and with multiple biological pathways involved.
- Genetic screening, while attractive, may not be proven fruitful.

### Generating Novel Vascularized Organoids for Disease Modeling and Drug Development

The advent of organoids, bioprinting, and organ-on-a-chip technologies has at long last offered viable alternatives to simplistic in vitro models and nonhuman in vivo approaches. 226 227 228 Species and tissue-specific three-dimensional (3D) cultures which mimic the architectural, molecular, and cellular complexity of human organs (to varying degrees) offer many of the benefits of in vitro systems (scalability, manipulability, etc.). They have demonstrated remarkable utility in drug screening, the generation of patient-specific and precision medicine models, and are allowing for unique insights into how cells interact with one another in complex 3D structures. 226

During the SARS-CoV-2 pandemic, organoid models demonstrated their utility in investigating poorly understood aspects of disease pathology. Our own work using microvascular organoid models demonstrated an important role of pericyte-mediated viral uptake in the loss of vascular integrity contributing to thrombosis in severe COVID-19 infection. 229 More recently, a vascularized BM organoid was developed and validated which faithfully recapitulates key features of the myelopoietic central BM. It was demonstrated that this system allows for drug screening in the context of myelofibrosis, but more importantly supports the engraftment of primary patient cells from several cancers which have been classically difficult to study ex vivo (primary myelofibrosis, multiple myeloma, acute lymphocytic leukemia). 230

With the promise of these approaches in mind, they are not without their limitations. Self-arranging organoids, particularly those derived from human-induced pluripotent stem cells, remain relatively fetal in their development, and engineering more “adult” versions of these systems remains a key area of study and improvement. While “organ-on-a-chip” and bioprinting strategies offer the promise of mimicking more adult tissue, they do so at the cost of the scalability and accessibility of these models. Moreover, cost remains a significant factor in the generation of certain organoid systems.

As the tissue engineering field continues to grow and expand, a key consideration is interpreting data derived from these models. While most researchers would balk at the notion of completely replacing animal systems with 3D human models, this is ultimately the end goal of many who are working in the field.

Key questions remain: how to reconcile conflicting human and murine data? How to meaningfully interpret mechanistic information in a (still) artificial system? These and other considerations are, and should be, part of the on-going dialogue between basic scientists, engineers, and clinicians about meaningfully exploiting what promise to be revolutionary approaches to how to model disease and develop therapies.

Potential areas for future investigation:

- Organoids are an important advance that will enrich the drug discovery process, alongside the use of current assays/mouse models.
- Organoids could be used as part of an iterative approach, with simpler organoid models used in screening before moving on to more complex systems.
- Use of organoids to instruct choice of drug in personalized medicine approaches is challenging and currently unproven, but with future developments could be feasible and valuable.

## Theme 5: Clinical Dilemmas in Thrombosis and Antithrombotic Management

### New Insights into Inherited Thrombophilia

The association between inherited thrombophilia and the occurrence of (recurrent) VTE has been demonstrated in the past focusing only on a few genetic defects including antithrombin (AT), protein C, protein S deficiencies and two polymorphisms, factor V Leiden (FVL) and prothrombin G20210A mutations. 231 Surprisingly, the vast majority of information clinicians daily use for the management of thrombophilic patients is based on the results of previous studies only dealing with thrombophilia mechanisms discovered in the second half of the last century. In contrast, it is commonly seen that in a large number (almost 50%) of families symptomatic for thrombophilia, none of these defects can be identified. The logical consequence is that other still unknown inherited thrombophilia may exist. Recently, new genetic defects responsible for severe thrombophilia have been identified, namely, pseudo-homozygosity for aPC resistance, the hyperfunctional FIX and FVIII, and the resistance to AT. 231

FVL is responsible for approximately 95% of cases of APC resistance. However, several point mutations in the F5 gene causing APC resistance have been identified in different populations. 231 Recently, severe thrombophilia in a factor V-deficient patient homozygous for the Ala2086Asp mutation (FV Besançon) has been described that affects anticoagulant pathways more strongly than the prothrombinase activity of FVa. 232 It can also occur that heterozygous FVL carriers present with a concomitant heterozygous F5 gene mutation responsible for FV deficiency, resulting in the 50% of FV plasma levels being all FVL. In these pseudo-homozygotes the thrombotic risk is as high as that observed in homozygous individuals. 233

Factor IX Padua is a gain-of-function mutation in the F9 gene (R338L) discovered in 2009 detected in a family symptomatic for VTE and exhibiting extremely high plasma factor IX activity (eight times the normal) with concomitant normal antigen levels. 234 Very recently, another hyper-functional FIX variant (R338Q, Factor IX Shanghai) was identified in a 13-year old boy referred for recurrent deep vein thrombosis (DVT). 235 In 2021 the first thrombophilic defect in the F8 gene (FVIII Padua) associated with markedly elevated FVIII levels and severe thrombophilia was described in two Italian families. 236 Genetic analysis revealed a 23.4-kb tandem duplication of the proximal portion of the F8 gene (promoter, exon 1, and a large part of intron 1), which co-segregated with high FVIII levels in the family. Finally, in 2012 a novel gain-of-function polymorphism leading to resistance to AT has been identified. 237 The molecular basis is a missense mutation of the prothrombin Arg596 residue (exon 14) resulting in impaired thrombin–AT binding and defective inhibition of the mutated thrombin by AT. Other similar cases were subsequently described in Serbia, India, and Italy. The symptomatic five families show three different mutations of the Arg596, and namely: prothrombin Yukuhashi Arg596Leu, 237 prothrombin Belgrade and Amrita Arg596Gln, 238 239 and prothrombin Padua 2 Arg596Trp. 240 Although all these hereditary thrombophilias are rare, clinicians ought to keep in mind these novel mutations when dealing with patients or families with unexplained history of recurrent VTE. Nonetheless, the large number of newly discovered inherited defects in the last decades seems to justify why one should not abandon testing for thrombophilia patients belonging to families with VTE.

In fact, previous epidemiological studies and recommendations are based on limited knowledge of inherited thrombophilic conditions. Advanced diagnostic tools including NGS are now adding important information on the etiology of thrombosis. Thus, new clinical studies are needed to re-define the role of inherited thrombophilia in the management of patients with thrombosis.

### Managing Atrial Fibrillation in Hemophilia

In the community of patients with hemophilia (PWH), cardiovascular disease is an emerging medical issue as the lifespan of these individuals continues to approach that of the general population. 241 A specific topic concerns patients with AF, where anticoagulants are widely used for the prevention of IS and systemic embolism.

The overall prevalence of AF in PWH in Europe is 0.84% and increases to 3.4% in patients >60 years and is therefore not different from that in the general population. 242 In a patient with a congenital bleeding disorder such as hemophilia, the decision to start antithrombotic therapy is even more challenging as the balance between thrombosis and hemorrhage is quite delicate.

In PWH with AF, there are many uncertainties to deal with by clinicians in clinical-decision making. First, the minimum clotting level to be able to start anticoagulation therapy is unknown. Several experts and consensus statements suggest that a minimum factor VIII/IX level of 20 to 30 IU/dL is needed for oral anticoagulation 241 242 243 244 245 and this is somewhat confirmed by a clinical registry. 246 On the other hand, PWH with factor levels <20 IU/dL might be considered naturally anticoagulated, as depicted by lower endogenous thrombin potential levels. 247

In the general population with AF, a risk score, such as the CHA _2_ DS _2_ -VASc score, is used to identify patients at risk for IS and therefore in need for anticoagulation therapy. In addition, the HAS-BLED score has been used to predict bleeding events on oral anticoagulation therapy. Balancing these two scores helps the clinician to decide whether the downside of oral anticoagulation outweighs the prevention of thrombotic events. However, in PWH these scores have not been and probably never will be prospectively validated due to the low number of adverse events in this specific population. Therefore, due to lack of evidence, treatment of PWH with AF should always be individualized taking into account the bleeding and thrombotic risk. As a general thought, PWHs with factor levels <20 IU/dL probably do not need additional antithrombotic therapy. In patients with mild hemophilia (>20 IU/dL), oral anticoagulation therapy is probably feasible. In that case, a DOAC has the preference over VKAs due to their favorable safety profile. 248

There is a strong need for more clinical data on anticoagulation therapy in PWH. Ideally, a registry is started to document the efficacy and safety of different types of antithrombotic treatment in PWH. However, due to the low event rates this will be a difficult task. Furthermore, there is a need for clinical validation of global hemostatic assays or thrombin generation tests to adapt individualized treatments. Especially, with the rapid adaptation of nonfactor replacement therapies (i.e., emicizumab), our long-lasting experience with factor levels will be challenged and the need for these hemostatic tests will be increasing.

### The Elusive Safe Antiplatelet Agent

Platelets are activated by two major groups of receptors, G protein-coupled receptors, which are the targets for current antiplatelet drugs, and tyrosine kinase-linked receptors, which are targets for a new class of antiplatelet agents. All of the current antiplatelet drugs increase the risk of bleeding and this can give rise to nuisance bleeds that may influence compliance and, in a minority of patients, life-threatening bleeds. Furthermore, over 50% of patients on antiplatelet medication experience further thrombotic episodes. Thus, there is an urgent need for drugs with improved efficacy that spare hemostasis.

The last major, widely prescribed new class of antiplatelet drugs introduced into the clinic was that of the P2Y _12_ receptor antagonists over 20 years ago, with the thienopyridine, clopidogrel, being the first in class. Several other P2Y _12_ receptor antagonists have since been introduced of which ticagrelor is the most notable because of its reversible action and greater efficacy. This offers an advantage over the irreversible thienopyridines but at the risk of increased bleeding. A PAR1 thrombin receptor antagonist, vorapaxar, has also been introduced but has not been widely described due to the increase in risk of bleeding.

The major tyrosine kinase-linked receptors in terms of signal strength are those with a motif in their cytosolic tail known as an immunoreceptor tyrosine-based activation motif (ITAM). Human platelets express three ITAM receptors, CLEC-2, GPVI and FcγRIIA, and all three signal through Src, Syk, and Btk tyrosine kinases. However, within this group, only the collagen and fibrin(ogen) receptor GPVI has been shown to play a role in hemostasis, although the importance of this appears to have been overestimated. This is shown by clinical data on patients in Chile with an insertion mutation that introduces a stop codon prior to the transmembrane sequence of GPVI and thus prevents surface expression. It is estimated that over 4,000 individuals are homozygous for loss of GPVI in Chile and yet only 12 cases from 11 unrelated families have been found. 249 The majority of these have a mild bleeding diathesis which in some cases has diminished/disappeared on reaching adulthood. Furthermore, only two patients with an inherited deficiency in GPVI have been reported outside of Chile. Given that collagen is a standard agonist in the clinic for the study of patients with a suspected platelet disorder, these data suggest that loss of GPVI does not give rise to a major bleeding diathesis.

This conclusion is also supported by a phase I safety trial on a GPVI-blocking Fab, now known as glenzocimab. 250 A press release in February 2022 on a Phase Ib and IIa trial on glenzocimab reported a tendency to a reduction in bleeding and improvement in cognitive symptoms in patients with acute IS when given in combination with standard treatment (thrombolysis or thrombectomy). This study was powered for safety rather than efficacy but the observation of an encouraging therapeutic effect provides a basis for a phase III trial and reinforces GPVI as a target for a new class of antiplatelet drugs.

This safe targeting of GPVI in terms of bleeding is further supported by clinical data on the use of Btk and Syk kinase inhibitors in the treatment of B cell malignancies and immune thrombocytopenia (ITP). In both cases, the bleeding symptoms reduce over time showing that ITAM-based signaling pathways can be safely targeted (in terms of bleeding) with kinase inhibitors even when the starting platelet count is thrombocytopenic. Inhibitors of Src, Syk, and Btk tyrosine kinases have been introduced into the clinic for treatment of these disorders and have been shown to be well tolerated for up to several years. Moreover, these inhibitors target activation of platelets by all their ITAM receptors. The first-generation inhibitor of the Tec family kinase Btk, ibrutinib, was shown to cause excessive bleeding raising concerns about its use as an antiplatelet drug, but this is now recognized to be due to one or more off-target effects, most likely on other kinases. The second- and third-generation inhibitors of Btk, such as acalabrutinib, and the Syk inhibitor, fostamatinib, have been shown to be well tolerated in patients, with bleeding symptoms reducing over time as patients respond to treatment. This is particularly notable for fostamatinib which is used in patients with refractory ITP and who therefore have a low platelet count. 251

The C-type lectin-like receptor CLEC-2 appears to have little or no role in hemostasis in humans and an uncertain role in arterial thrombosis. In contrast, CLEC-2 has been shown to drive thrombosis at sites of inflammation in the venous system in mouse thrombo-inflammatory models, namely DVT and bacterial infection. 252 Platelet activation in these models is mediated by inflammation-driven up-regulation of the ligand for CLEC-2 in the vessel wall podoplanin. Patients treated with ibrutinib show a reduction in DVT suggesting that CLEC-2 may also drive thrombosis in thrombo-inflammatory disease in humans. 253

The low affinity immune receptor FcγRIIA is the only Fc receptor on platelets and has no known role in hemostasis. Activation of FcγRIIA underlies heparin-induced thrombocytopenia (HIT) which is associated with a marked reduction in platelet count and in some patients life-threatening thrombosis. The molecular basis of this disorder is the formation of antibodies that bind to the positively charged chemokine PF4 which forms an immune complex with the negatively charged heparin. A related, but much rarer condition, with less than 50 cases world-wide, autoimmune HIT, is also mediated by anti-PF4 antibodies but is independent of heparin. In February 2021, the first cases of a new syndrome, now known as vaccine-induced immune thrombocytopenia and thrombosis (VITT), were identified in patients who had received a first dose of the Oxford-AZ adenovirus vaccine to SARS-COV-2 in the previous 5 to 20 days. The frequency of VITT is extremely low, in the order of 1:50,000 to 100,000. VITT is also mediated by antibodies to platelet factor 4 (PF4), with the binding of PF4 to the adenovirus vector driving antibody production. 254 Platelet activation by sera from patients with VITT can be prevented by treatment with a Src, Syk, or Btk inhibitor, although the low frequency of the syndrome, cost of the kinase inhibitors, and potential side-effects prevent this being translated to a clinical trial. 255

In summary, platelet tyrosine kinase-linked receptors, notably glycoprotein VI (GPVI), represent targets for a new class of antiplatelet drugs that may be more powerful against arterial thrombotic disorders such as ACSs and IS than current drugs with a reduce risk of bleeding. In addition, they are targets in both thrombo-inflammatory disorders and immune complex-driven thrombosis, two groups which are not currently treated with antiplatelets. Receptor blockade can be achieved using protein-based inhibitors such as the GPVI Fab glenzocimab or small-molecule inhibitors targeted to Src, Syk, or Btk tyrosine kinases. Potent small-molecule inhibitors of GPVI, CLEC-2, and FcγRIIA have not been identified. The kinase inhibitors have the advantage of being orally available and blocking activation by all three ITAM receptors but with the concern of off-target effects on myeloid cells and lymphocytes leading to an increase in susceptibility to infection. The irreversible nature of the second- and third-generation Tec family kinase inhibitors such as rilzabrutinib may enable them to be used at a much lower concentration thus reducing off-target effects. 256

### Will FXIa Inhibition Fulfill a Promise?

#### FXI Deficiency (Hemophilia C or Rosenthal Disease)

In 1953 Rosenthal et al described this autosomal disorder in a family with bleeding events during surgery or dental procedures. The prevalence of severe FXI deficiency is about ≈1/million, and more frequent in certain populations. Clinically, the prolongation of the aPTT may lead to the diagnosis, rather than bleeding complications, which are generally mild, even in severe deficiency. Bleeding may be provoked by surgery, particularly in tissues with high fibrinolytic activity like urogenital or oropharyngeal, but may also include epistaxis, heavy menstrual bleeding, or postinjury, while unprovoked bleeding into muscle or soft tissue or hemarthrosis is not frequent.

Bleeding may also occur in heterozygous subjects with mild deficiency (20–60%) and does not correlate with FXI level. Bleeding can be corrected by FXI (blood product or recombinant clotting factor). Pronounced FXI deficiency lowers risk for IS and venous thrombosis.

#### FXI(a) Inhibition

FXI(a) is therefore an interesting target for antithrombotic therapy as upstream inhibition of the intrinsic cascade may be effective, yet potentially safer with regard to bleeding as FXI-deficient patients rarely have spontaneous bleeding, suggesting that FXI may have a limited role in hemostasis. FXI ^−/−^ mice have normal tail bleeding times but show decreased clot formation at injury sites of arterial or venous. 257 Likewise, treatment of rodent or rabbit models with FXI antisense oligonucleotides (FXI-ASO) or anti-FXI antibodies has shown resistance to experimentally induced thrombosis and a low risk of bleeding complications. 258 259 Different strategies targeting FXI/FXIa for antithrombotic therapy are under development in clinical trials. Novel FXI inhibitor agents include inhibitors of biosynthesis, antibodies, and small molecules ( Table 4 ).

**Table 4:** Factor XI(a) inhibition

	Type of FXI inhibition	Administration	Frequency	Onset of action	Offset of action	Renal excretion
ASOs	Block biosynthesis	Parenteral	Weekly to monthly	Slow (weeks)	Slow (weeks)	No
Antibodies	Bind target protein	Parenteral	Monthly	Rapid (hours to days)	Slow (weeks)	No
Small molecules	Bind target protein	Oral (or parenteral)	Daily	Rapid (minutes to hours)	Fast	Yes
Natural inhibitors	Bind target protein	Parenteral	Daily	Rapid (minutes)	Fast	Uncertain
Aptamers	Bind target protein	Parenteral	Daily	Rapid (minutes to hours)	Fast	No

#### The Clinical Trials of Targeting FXI

Four FXI(a) inhibitors have been tested in patients undergoing total knee arthroplasty (TKA). FXI ASO IONIS-FXIRX that inhibits FXI biosynthesis in liver and abelacimab (MAA868) that inhibits FXI by binding the catalytic domain of both FXI (zymogen) and FXIa were compared with enoxaparin (40 mg) for prevention of VTE in TKA patients. In the FXI ASO trial, the study showed that the higher dose (300 mg) regimen (4%) was superior to enoxaparin (30%) for the prevention of VTE and had a lower rate of bleeding events than with enoxaparin. 260 Similar to the FXI ASO result, the trial of abelacimab showed that the incidence of VTE in the 30 mg abelacimab regimen was noninferior to enoxaparin, and the 75 and 150 mg abelacimab regimens were superior to enoxaparin ( *p* < 0.001). 261

Osocimab (BAY 1213790), a monoclonal antibody that can inhibit FXIa, was tested in 813 adult TKA patients (FOXTROT). Osocimab (0.6, 1.2, and 1.8 mg/kg) was compared with enoxaparin and apixaban for thromboprophylaxis, and was noninferior with respect to efficacy, while it caused less bleeding. 262 Likewise, milvexian, a small molecule that inhibits FXIa activity, was effective for the prevention of VTE and was associated with a low risk of bleeding when compared with enoxaparin at five different dosing regimens . Hence, these trials demonstrated that FXI contributes to postoperative VTE and that lowering FXI levels or inhibiting its activity provides an effective and possibly safe method for its prevention.

For the patient with AF, abelacimab (120 mg, 180 mg) (NCT04213807) and the small molecule asundexian (BAY 2433334) (PACIFIC-AF, NCT04218266) are compared with placebo or apixaban. The first phase 2b trial data of PACIFIC-AF have been already published. Compared with apixaban in patients with AF at risk of stroke, the bleeding rate for the primary endpoint (ISTH major and clinically relevant nonmajor bleeding) was reduced by 67% in patients receiving asundexian. 49 263 However, PACIFIC-AF was not powered to test differences in rates of thrombotic events between groups. There are another two different phase II clinical trials in which asundexian was tested: PACIFIC AMI and PACIFIC Stroke, which were both recently published. In patients with ACS (NSTEMI and STEMI), asundexian on top of DAPT (ASS plus any P2Y12 inhibitor) resulted in dose-dependent, near-complete inhibition of FXIa activity without a significant increase in bleeding and a low rate of ischemic events when compared with DAPT alone. 264 In patients with noncardioembolic IS, asundexian on top of single antiplatelet therapy did not increase the risk of major bleeding, but did also not reduce the composite of covert brain infarction or IS. 265 In the Axiomatic trial, the safety of milvexian, another direct FXIa inhibitor, was tested in noncardioembolic stroke compared with placebo. Similarly, this trial did not show significantly increased bleeding compared with placebo, without having the power to assess efficacy. Taken together, these findings warrant further investigation in phase III clinical trials. The OCEANIC AF (NCT02168829) is the first of its kind to test efficacy of asundexian as compared with apixaban in AF.

#### Bleeding Management of FXI Deficiency and FXI Inhibition

For the clinical use of FXI inhibitors—not only those with the long half-life—the management of bleeding or peri-procedural management is crucial. Bleeding management in patients with FXI deficiency includes fresh frozen plasma, FXI concentrates (half-life 50–70 hours), which may be administered every 48 to 72 hours, also low-dose rFVIla (e.g., lower doses of rFVlla [15–20 µg/kg]), and antifibrinolytic agents, such as tranexamic acid. Antithrombotic agents, such as anticoagulants and antiplatelet medications, should generally be avoided.

Reversal studies of FXI inhibitors are being performed in healthy volunteers using PCC and rFVIla, and fully human antibody Fab fragments with very high affinity for FXIa inhibitors are being explored for their potential to neutralize their anticoagulant effects.

#### Outlook for FXI Inhibition

The pathophysiologic concept of FXI inhibition with separating thrombosis from bleeding is very promising and supported by the clinical presentation of FXI deficiency patients and animal models. In addition, FXI inhibition also links to inflammatory pathways and with the contact pathway may also be an effective antithrombotic treatment for foreign surfaces.

However, the benchmark of today's anticoagulant treatment achieved with DOACs is not easily surpassed. Therefore, identifying the important medical needs, selecting the appropriate indications, and choosing the optimal trial design will determine the future success of FXI inhibition. Potential other areas of interest are patients with cancer and thrombosis and patients with severe renal insufficiency or other factors that are associated with high risks for bleeding (and thrombosis).

### How to Prevent Thrombosis in the Next Corona Pandemic; Lessons Learned

COVID-19 brought the clinical and research world into widespread recognition of the problem of coagulopathy in infections. Very early identification of thrombosis in patients with COVID-19 first reported from China paved the way for the publication of a flurry of guidelines focused on the antithrombotic management of these patients. 266 Soon after, the research world started turning their attention to the mechanisms of thrombosis in COVID-19 and how the different pathways may be involved in the thrombotic complication. 267 In the Maastricht discussion, several clinical pointers were presented to assist in future management of hemostatic and thrombotic complications associated with infections. In COVID-19, the preponderance of thrombosis is in the pulmonary circulation. 268 This should ideally be termed as pulmonary thrombosis rather than pulmonary emboli. The rationale for this consideration is the activation of localized bronchoalveolar coagulation by the SARS-CoV-2 virus and the hosts' immune system (widely known as immunothrombosis) in the causation of these clots. These are different to the emboli from lower limbs or other parts of the circulatory beds, which are commonly recognized as pulmonary emboli. The presence of localized coagulation systems may occur in the gastrointestinal tract and possibly the integumental barrier or at sites where pathogen entry is likely. Moving on to the laboratory aspects, D-dimer elevation is a characteristic aspect of COVID-19. 269 This is predominantly due to alveolar fibrinolysis rather than clot breakdown and hence correlated with prognosis in these patients rather than with thrombotic risks. A useful future study would be to look at how extravascular fibrinolysis may correlate with disease outcomes in different pathological states. Severe thrombocytopenia is rare in COVID-19 although mild to moderate drop in platelet counts can be common. 270 There are also reports of markedly elevated vWF levels in these patients too, which in some reports were correlating strongly with poor outcomes. Can these two be linked? Possibly, the thrombocytopenia is caused by the release of large amounts of vWF from endothelial activation which means a decrease of platelet counts can be suggestive of microthrombus formation. This leads to under-recognition of microvascular thrombi from a clinical point of view. In the absence of other clear explanations, a drop in the platelet counts or fibrinogen levels in the setting of sepsis or inflammatory states may mean formation of microthrombi and the need for intervention to limit this process. But the timing of intervention is important too. Coagulation systems including platelets and fibrinogen are anti-infective and as such are commonly activated in different infections. There is a fine line between these beneficial effects of the host's hemostatic system turning to the harmful state of micro- and macrovascular thrombosis. The ideal time for intervention is that period when the shift to harm from a beneficial period occurs. 271 Monitoring trends in the common tests may be the way forward in this regard but future research should also focus on the different pathways and the correct timing for intervention targeting the coagulation system in infections.

Areas of potential research with the lessons learnt from the corona pandemic include: (1) the importance of differentiating localized thrombosis from systemic coagulation activation and how we can target site-specific thrombosis and thus minimize bleeding from systemic antithrombotic therapy; (2) examination of how extravascular fibrinolysis may correlate with disease outcomes in different pathological states; (3) how the trends in laboratory markers may guide treatment decisions to escalate or withdraw antithrombotic agents, and (4) what may be the best time and pathways to target the activated platelets and coagulation system for host benefit.

### TICARDIO Translational Lecture: Ambivalent Role of Leukocyte-Derived Microvesicles in Hemostasis

Microvesicles, resulting from vascular and blood cell activation, are now recognized as new protagonists in cellular crosstalk involved in thrombo-inflammation.

Initially described as catalytic surfaces able to activate TF-dependent procoagulant pathways, leukocyte-derived microvesicles (LMVs) were more recently ascribed a fibrinolytic activity. 272 273 Using first whole blood stimulated with LPS (LPS-MV) to mimic inflammatory conditions, granulocyte MVs were found to lyse a thrombus in vitro, according to their plasmin generation capacity (MV-PGC), in a uPA/uPAR-dependent manner. 274 Second, defining MV coagulolytic balance (MV-CLB) as the ratio between MV procoagulant and fibrinolytic activity, the impact of MV with distinct CLB profile was investigated on the dynamics of thrombus formation in vivo, using a laser injury model of mice arterial thrombosis and intravital microscopy. Interestingly, plasminogen accumulation reflecting fibrinolysis initiation was higher in mice receiving fibrinolytic EV-BCL compared with procoagulant EV-CLB profile.

Accumulated knowledge on the role of LMV has not only revisited their role as ambivalent catalytic surfaces able to tune a coagulolytic balance 275 276 277 but have also driven technological advances, resulting in the development of sensitive and specific assays allowing the measurement of MV-driven TF procoagulant and plasmin fibrinolytic activity. 278 279 280

According to TICARDIO objectives on new pathways and targets involving LMV in immuno-thrombotic responses and their translation into novel diagnostic and therapeutic strategies, sepsis-induced coagulopathy was chosen as a typical thrombo-inflammatory clinical situation associating coagulation activation and abnormal fibrinolysis. While converging animal and clinical studies emphasized the deleterious role of procoagulant MV in sepsis and septic shock, the hypothesis was that MVs have a protective effect supported by their capacity to lyse a thrombus. Granulocyte MVs from sepsis patients were found to display a heterogeneous pattern of PGC, driven by uPA-uPAR expression, and were able to lyse a thrombus according to their MV-PGC level. Injection of granulocyte MV with a high PGC level reduced clot formation and improved survival in a mouse model of septic shock, demonstrating a protective effect of these granulocytic subpopulations, 274 opening perspectives for a potential antithrombotic strategy. In a cohort of 225 patients with septic shock enrolled in a multicenter prospective study, the MV-CLB predicted mortality in septic shock patients with better performances than the procoagulant and profibrinolytic activities taken individually, and allowed stratifying the severity of septic shock . This new functional signature of MV opens unexplored avenues for the guidance of individualized therapy targeting coagulopathy in septic shock.

Data presented in the SEPSIS context illustrate the view of granulocyte MV-CLB as an ambivalent microsystem tuning thrombo-inflammation.

Potential areas for future investigation:

- From one side, a deeper understanding of what determines the MV-CLB, including the role of the distinct triggers and subsets of MV and the impact of pharmacological modulations, is required.
- From another side, the definition of the true value of MV as biomarkers of thrombotic risk, through multicenter prospective clinical studies thanks to methodological innovation and standardization, to measure MV in a more automatized way and integrate them into scoring systems with other biomarkers and clinical variables. These perspectives are included in ongoing research programs. 125

## References

[1] Ramcharan K S Lip G YH Stonelake P S Blann A D The endotheliome: a new concept in vascular biology Thromb Res 2011 128 01 1 7 21168189 10.1016/j.thromres.2010.11.019

[2] Stroke Experts Collaboration Group Owolabi M O Thrift A G Mahal A Primary stroke prevention worldwide: translating evidence into action Lancet Public Health 2022 7 01 e74 e85 34756176 10.1016/S2468-2667(21)00230-9 PMC8727355

[3] Kamarova M Baig S Patel H Antiplatelet use in ischemic stroke Ann Pharmacother 2022 56 10 1159 1173 35094598 10.1177/10600280211073009 PMC9393649

[4] Serebruany V L Kim M H Hanley D F Vorapaxar monotherapy for secondary stroke prevention: a call for randomized trial Int J Stroke 2016 11 06 614 617 26860124 10.1177/1747493016632253

[5] De Luca C Colangelo A M Alberghina L Papa M Neuro-immune hemostasis: homeostasis and diseases in the central nervous system Front Cell Neurosci 2018 12 459 30534057 10.3389/fncel.2018.00459 PMC6275309

[6] Sokolova E Reiser G Prothrombin/thrombin and the thrombin receptors PAR-1 and PAR-4 in the brain: localization, expression and participation in neurodegenerative diseases Thromb Haemost 2008 100 04 576 581 18841278

[7] Von dem Borne P A Bajzar L Meijers J C Nesheim M E Bouma B N Thrombin-mediated activation of factor XI results in a thrombin-activatable fibrinolysis inhibitor-dependent inhibition of fibrinolysis J Clin Invest 1997 99 10 2323 2327 9153272 10.1172/JCI119412 PMC508069

[8] Suri M FK Yamagishi K Aleksic N Hannan P J Folsom A R Novel hemostatic factor levels and risk of ischemic stroke: the Atherosclerosis Risk in Communities (ARIC) Study Cerebrovasc Dis 2010 29 05 497 502 20299790 10.1159/000297966 PMC2865476

[9] Salomon O Steinberg D M Koren-Morag N Tanne D Seligsohn U Reduced incidence of ischemic stroke in patients with severe factor XI deficiency Blood 2008 111 08 4113 4117 18268095 10.1182/blood-2007-10-120139

[10] Undas A Slowik A Gissel M Mann K G Butenas S Circulating activated factor XI and active tissue factor as predictors of worse prognosis in patients following ischemic cerebrovascular events Thromb Res 2011 128 05 e62 e66 21820158 10.1016/j.thromres.2011.06.010 PMC3205247

[11] Bouma B N Mosnier L O Meijers J C Griffin J H Factor XI dependent and independent activation of thrombin activatable fibrinolysis inhibitor (TAFI) in plasma associated with clot formation Thromb Haemost 1999 82 06 1703 1708 10613658

[12] Leung P Y Hurst S Berny-Lang M A Inhibition of factor XII-mediated activation of factor XI provides protection against experimental acute ischemic stroke in mice Transl Stroke Res 2012 3 03 381 389 23634198 10.1007/s12975-012-0186-5 PMC3637928

[13] Maroney S A Westrick R J Cleuren A C Tissue factor pathway inhibitor is required for cerebrovascular development in mice Blood 2021 137 02 258 268 32735640 10.1182/blood.2020006054 PMC7820871

[14] Ginhoux F Greter M Leboeuf M Fate mapping analysis reveals that adult microglia derive from primitive macrophages Science 2010 330(6005):841 845 20966214 10.1126/science.1194637 PMC3719181

[15] Hollister R D Kisiel W Hyman B T Immunohistochemical localization of tissue factor pathway inhibitor-1 (TFPI-1), a Kunitz proteinase inhibitor, in Alzheimer's disease Brain Res 1996 728 01 13 19 8864292

[16] Ziliotto N Bernardi F Jakimovski D Hemostasis biomarkers in multiple sclerosis Eur J Neurol 2018 25 09 1169 1176 29758118 10.1111/ene.13681

[17] Ziliotto N Lamberti N Manfredini F Functional recovery in multiple sclerosis patients undergoing rehabilitation programs is associated with plasma levels of hemostasis inhibitors Mult Scler Relat Disord 2020 44 102319 32593960 10.1016/j.msard.2020.102319

[18] Yang L Li Y Bhattacharya A Zhang Y A plasma proteolysis pathway comprising blood coagulation proteases Oncotarget 2016 7 27 40919 40938 27248165 10.18632/oncotarget.7261 PMC5173032

[19] Yang L Bhattacharya A Li Y Zhang Y Anticoagulants inhibit proteolytic clearance of plasma amyloid beta Oncotarget 2017 9 05 5614 5626 29464022 10.18632/oncotarget.23718 PMC5814162

[20] Zamolodchikov D Renné T Strickland S The Alzheimer's disease peptide β-amyloid promotes thrombin generation through activation of coagulation factor XII J Thromb Haemost 2016 14 05 995 1007 26613657 10.1111/jth.13209 PMC4870142

[21] Begic E Hadzidedic S Obradovic S Begic Z Causevic M Increased levels of coagulation factor XI in plasma are related to Alzheimer's disease diagnosis J Alzheimers Dis 2020 77 01 375 386 32804133 10.3233/JAD-200358

[22] AddNeuroMed Consortium Sattlecker M Kiddle S J Newhouse S Alzheimer's disease biomarker discovery using SOMAscan multiplexed protein technology Alzheimers Dement 2014 10 06 724 734 24768341 10.1016/j.jalz.2013.09.016

[23] Chen M Xia W Proteomic profiling of plasma and brain tissue from Alzheimer's disease patients reveals candidate network of plasma biomarkers J Alzheimers Dis 2020 76 01 349 368 32474469 10.3233/JAD-200110 PMC7457324

[24] Petersen M A Ryu J K Akassoglou K Fibrinogen in neurological diseases: mechanisms, imaging and therapeutics Nat Rev Neurosci 2018 19 05 283 301 29618808 10.1038/nrn.2018.13 PMC6743980

[25] Montagne A Nation D A Sagare A P APOE4 leads to blood-brain barrier dysfunction predicting cognitive decline Nature 2020 581(7806):71 76 32376954 10.1038/s41586-020-2247-3 PMC7250000

[26] Ryu J K Rafalski V A Meyer-Franke A Fibrin-targeting immunotherapy protects against neuroinflammation and neurodegeneration Nat Immunol 2018 19 11 1212 1223 30323343 10.1038/s41590-018-0232-x PMC6317891

[27] Ahn H J Glickman J F Poon K L A novel Aβ-fibrinogen interaction inhibitor rescues altered thrombosis and cognitive decline in Alzheimer's disease mice J Exp Med 2014 211 06 1049 1062 24821909 10.1084/jem.20131751 PMC4042638

[28] Chen Z-L Revenko A S Singh P MacLeod A R Norris E H Strickland S Depletion of coagulation factor XII ameliorates brain pathology and cognitive impairment in Alzheimer disease mice Blood 2017 129 18 2547 2556 28242605 10.1182/blood-2016-11-753202 PMC5418637

[29] Shnerb Ganor R Harats D Schiby G Elderly apolipoprotein E–/– mice with advanced atherosclerotic lesions in the aorta do not develop Alzheimer's disease-like pathologies Mol Med Rep 2018 17 02 2488 2492 29207114 10.3892/mmr.2017.8127

[30] Shnerb Ganor R Harats D Schiby G Factor XI deficiency protects against atherogenesis in apolipoprotein E/factor XI double knockout mice Arterioscler Thromb Vasc Biol 2016 36 03 475 481 26800563 10.1161/ATVBAHA.115.306954 PMC4785893

[31] Ngo A TP Jordan K R Mueller P A Pharmacological targeting of coagulation factor XI mitigates the development of experimental atherosclerosis in low-density lipoprotein receptor-deficient mice J Thromb Haemost 2021 19 04 1001 1017 33421301 10.1111/jth.15236 PMC8549080

[32] Kaikita K Takeya M Ogawa H Suefuji H Yasue H Takahashi K Co-localization of tissue factor and tissue factor pathway inhibitor in coronary atherosclerosis J Pathol 1999 188 02 180 188 10398162 10.1002/(SICI)1096-9896(199906)188:2<180::AID-PATH338>3.0.CO;2-Q

[33] PRIME Study Group Morange P E Simon C Alessi M C Endothelial cell markers and the risk of coronary heart disease: the Prospective Epidemiological Study of Myocardial Infarction (PRIME) study Circulation 2004 109 11 1343 1348 15023872 10.1161/01.CIR.0000120705.55512.EC

[34] Figueras J Monasterio J Lidón R M Sambola A Garcia-Dorado D Lower tissue factor inhibition in patients with ST segment elevation than in patients with non ST elevation acute myocardial infarction Thromb Res 2012 130 03 458 462 22424853 10.1016/j.thromres.2012.02.044

[35] Atherogene Investigators Morange P E Blankenberg S Alessi M C Prognostic value of plasma tissue factor and tissue factor pathway inhibitor for cardiovascular death in patients with coronary artery disease: the AtheroGene study J Thromb Haemost 2007 5 03 475 482 17204132 10.1111/j.1538-7836.2007.02372.x

[36] Zhao Y Yu Y Shi M Association study to evaluate TFPI gene in CAD in Han Chinese BMC Cardiovasc Disord 2017 17 01 188 28716011 10.1186/s12872-017-0626-y PMC5514508

[37] Naji D H Tan C Han F Significant genetic association of a functional TFPI variant with circulating fibrinogen levels and coronary artery disease Mol Genet Genomics 2018 293 01 119 128 28894953 10.1007/s00438-017-1365-6 PMC5794607

[38] Opstad T B Pettersen A A Weiss T Arnesen H Seljeflot I Gender differences of polymorphisms in the TF and TFPI genes, as related to phenotypes in patients with coronary heart disease and type-2 diabetes Thromb J 2010 8 7 20444258 10.1186/1477-9560-8-7 PMC2882354

[39] Moatti D Haidar B Fumeron F A new T-287C polymorphism in the 5′ regulatory region of the tissue factor pathway inhibitor gene. Association study of the T-287C and C-399T polymorphisms with coronary artery disease and plasma TFPI levels Thromb Haemost 2000 84 02 244 249 10959696

[40] Moatti D Seknadji P Galand C Polymorphisms of the tissue factor pathway inhibitor (TFPI) gene in patients with acute coronary syndromes and in healthy subjects : impact of the V264M substitution on plasma levels of TFPI Arterioscler Thromb Vasc Biol 1999 19 04 862 869 10195910 10.1161/01.atv.19.4.862

[41] Lorentz C U Verbout N G Cao Z Factor XI contributes to myocardial ischemia-reperfusion injury in mice Blood Adv 2018 2 02 85 88 29365314 10.1182/bloodadvances.2017004879 PMC5787867

[42] Kossmann S Lagrange J Jäckel S Platelet-localized FXI promotes a vascular coagulation-inflammatory circuit in arterial hypertension Sci Transl Med 2017 9(375):eaah4923 28148841 10.1126/scitranslmed.aah4923

[43] Preis M Hirsch J Kotler A Factor XI deficiency is associated with lower risk for cardiovascular and venous thromboembolism events Blood 2017 129 09 1210 1215 28039189 10.1182/blood-2016-09-742262

[44] Doggen C JM Rosendaal F R Meijers J CM Levels of intrinsic coagulation factors and the risk of myocardial infarction among men: opposite and synergistic effects of factors XI and XII Blood 2006 108 13 4045 4051 16931632 10.1182/blood-2005-12-023697

[45] Butenas S Undas A Gissel M T Szuldrzynski K Zmudka K Mann K G Factor XIa and tissue factor activity in patients with coronary artery disease Thromb Haemost 2008 99 01 142 149 18217146 10.1160/TH07-08-0499

[46] Salomon O Steinberg D M Dardik R Inherited factor XI deficiency confers no protection against acute myocardial infarction J Thromb Haemost 2003 1 04 658 661 12871398 10.1046/j.1538-7836.2003.00195.x

[47] Siegerink B Govers-Riemslag J WP Rosendaal F R Ten Cate H Algra A Intrinsic coagulation activation and the risk of arterial thrombosis in young women: results from the Risk of Arterial Thrombosis in relation to Oral contraceptives (RATIO) case-control study Circulation 2010 122 18 1854 1861 20956210 10.1161/CIRCULATIONAHA.110.943738

[48] Ząbczyk M T Hanarz M Malinowski K P Active FXI can independently predict ischemic stroke in anticoagulated atrial fibrillation patients: a cohort study Thromb Haemost 2022 122 08 1397 1406 35158400 10.1055/s-0042-1742366

[49] PACIFIC-AF Investigators Piccini J P Caso V Connolly S J Safety of the oral factor XIa inhibitor asundexian compared with apixaban in patients with atrial fibrillation (PACIFIC-AF): a multicentre, randomised, double-blind, double-dummy, dose-finding phase 2 study Lancet 2022 399(10333):1383 1390 35385695 10.1016/S0140-6736(22)00456-1

[50] Garlapati V Molitor M Michna T Targeting myeloid cell coagulation signaling blocks MAP kinase/TGF-β1-driven fibrotic remodeling in ischemic heart failure J Clin Invest 2023 133 04 e156436 36548062 10.1172/JCI156436 PMC9927945

[51] Aronovich A Nur Y Shezen E A novel role for factor VIII and thrombin/PAR1 in regulating hematopoiesis and its interplay with the bone structure Blood 2013 122 15 2562 2571 23982175 10.1182/blood-2012-08-447458 PMC5527398

[52] Borkowska S Suszynska M Mierzejewska K Novel evidence that crosstalk between the complement, coagulation and fibrinolysis proteolytic cascades is involved in mobilization of hematopoietic stem/progenitor cells (HSPCs) Leukemia 2014 28 11 2148 2154 24667943 10.1038/leu.2014.115 PMC4177021

[53] Gur-Cohen S Kollet O Graf C Regulation of long-term repopulating hematopoietic stem cells by EPCR/PAR1 signaling Ann N Y Acad Sci 2016 1370 01 65 81 26928241 10.1111/nyas.13013 PMC5193365

[54] Nguyen T S Lapidot T Ruf W Extravascular coagulation in hematopoietic stem and progenitor cell regulation Blood 2018 132 02 123 131 29866813 10.1182/blood-2017-12-768986 PMC6634957

[55] Gur-Cohen S Itkin T Chakrabarty S PAR1 signaling regulates the retention and recruitment of EPCR-expressing bone marrow hematopoietic stem cells Nat Med 2015 21 11 1307 1317 26457757 10.1038/nm.3960 PMC4776769

[56] Nevo N Zuckerman T Gur-Cohen S PAR1 expression predicts clinical G-CSF CD34 ^+^ HSPC mobilization and repopulation potential in transplanted patients HemaSphere 2019 3 04 e288 31942543 10.1097/HS9.0000000000000288 PMC6919473

[57] Nevo N Ordonez-Moreno L-A Gur-Cohen S Enhanced thrombin/PAR1 activity promotes G-CSF- and AMD3100-induced mobilization of hematopoietic stem and progenitor cells via NO upregulation Leukemia 2021 35 11 3334 3338 33654208 10.1038/s41375-021-01194-5

[58] Fares I Chagraoui J Lehnertz B EPCR expression marks UM171-expanded CD34 ^+^ cord blood stem cells Blood 2017 129 25 3344 3351 28408459 10.1182/blood-2016-11-750729

[59] Groeneweg K E Duijs J MGJ Florijn B W Circulating long noncoding RNA LNC-EPHA6 associates with acute rejection after kidney transplantation Int J Mol Sci 2020 21 16 5616 32764470 10.3390/ijms21165616 PMC7460577

[60] Humphreys B D Targeting pericyte differentiation as a strategy to modulate kidney fibrosis in diabetic nephropathy Semin Nephrol 2012 32 05 463 470 23062987 10.1016/j.semnephrol.2012.07.009 PMC3674859

[61] Lerman L O Chade A R Angiogenesis in the kidney: a new therapeutic target? Curr Opin Nephrol Hypertens 2009 18 02 160 165 19430335 10.1097/MNH.0b013e32831ec1db PMC2796055

[62] Bijkerk R Duijs J MGJ Khairoun M Circulating microRNAs associate with diabetic nephropathy and systemic microvascular damage and normalize after simultaneous pancreas-kidney transplantation Am J Transplant 2015 15 04 1081 1090 25716422 10.1111/ajt.13072

[63] Dólleman S C Agten S M Spronk H MH Thrombin in complex with dabigatran can still interact with PAR-1 via exosite-I and instigate loss of vascular integrity J Thromb Haemost 2022 20 04 996 1007 35037739 10.1111/jth.15642 PMC9306515

[64] Maroney S A Haberichter S L Friese P Active tissue factor pathway inhibitor is expressed on the surface of coated platelets Blood 2007 109 05 1931 1937 17082321 10.1182/blood-2006-07-037283 PMC1801047

[65] Uil M Hau C M Ahdi M Cellular origin and microRNA profiles of circulating extracellular vesicles in different stages of diabetic nephropathy Clin Kidney J 2019 14 01 358 365 33564439 10.1093/ckj/sfz145 PMC7857783

[66] Uil M Butter L M Claessen N Larsen P W Florquin S Roelofs J JTH Platelet inhibition by ticagrelor is protective against diabetic nephropathy in mice FASEB J 2020 34 10 13750 13761 32856376 10.1096/fj.202000897R

[67] Cameron-Vendrig A Reheman A Siraj M A Glucagon-like peptide 1 receptor activation attenuates platelet aggregation and thrombosis Diabetes 2016 65 06 1714 1723 26936963 10.2337/db15-1141

[68] Geovanini G R Libby P Atherosclerosis and inflammation: overview and updates Clin Sci (Lond) 2018 132 12 1243 1252 29930142 10.1042/CS20180306

[69] Hansson G K Libby P Tabas I Inflammation and plaque vulnerability J Intern Med 2015 278 05 483 493 26260307 10.1111/joim.12406 PMC5082111

[70] Quillard T Franck G Mawson T Folco E Libby P Mechanisms of erosion of atherosclerotic plaques Curr Opin Lipidol 2017 28 05 434 441 28682809 10.1097/MOL.0000000000000440 PMC5676466

[71] Randolph G J Mechanisms that regulate macrophage burden in atherosclerosis Circ Res 2014 114 11 1757 1771 24855200 10.1161/CIRCRESAHA.114.301174 PMC4059102

[72] Viola J Soehnlein O Atherosclerosis - a matter of unresolved inflammation Semin Immunol 2015 27 03 184 193 25865626 10.1016/j.smim.2015.03.013

[73] Bane C E Jr Ivanov I Matafonov A Factor XI deficiency alters the cytokine response and activation of contact proteases during polymicrobial sepsis in mice PLoS One 2016 11 04 e0152968 27046148 10.1371/journal.pone.0152968 PMC4821616

[74] Silasi R Keshari R S Regmi G Factor XII plays a pathogenic role in organ failure and death in baboons challenged with Staphylococcus aureus Blood 2021 138 02 178 189 33598692 10.1182/blood.2020009345 PMC8288658

[75] Silasi R Keshari R S Lupu C Inhibition of contact-mediated activation of factor XI protects baboons against *S aureus* -induced organ damage and death Blood Adv 2019 3 04 658 669 30808684 10.1182/bloodadvances.2018029983 PMC6391670

[76] Lorentz C U Tucker E I Verbout N G The contact activation inhibitor AB023 in heparin-free hemodialysis: results of a randomized phase 2 clinical trial Blood 2021 138 22 2173 2184 34086880 10.1182/blood.2021011725 PMC8641100

[77] Evans B R Yerly A van der Vorst E PC Inflammatory mediators in atherosclerotic vascular remodeling Front Cardiovasc Med 2022 9 868934 35600479 10.3389/fcvm.2022.868934 PMC9114307

[78] Jiang H Zhou Y Nabavi S M Mechanisms of oxidized LDL-mediated endothelial dysfunction and its consequences for the development of atherosclerosis Front Cardiovasc Med 2022 9 925923 35722128 10.3389/fcvm.2022.925923 PMC9199460

[79] Puy C Ngo A TP Pang J Endothelial PAI-1 (plasminogen activator inhibitor-1) blocks the intrinsic pathway of coagulation, inducing the clearance and degradation of FXIa (activated factor XI) Arterioscler Thromb Vasc Biol 2019 39 07 1390 1401 31242030 10.1161/ATVBAHA.119.312619 PMC6597189

[80] Endler G Marsik C Jilma B Schickbauer T Quehenberger P Mannhalter C Evidence of a U-shaped association between factor XII activity and overall survival J Thromb Haemost 2007 5 06 1143 1148 17388965 10.1111/j.1538-7836.2007.02530.x

[81] Cooley B C The dirty side of the intrinsic pathway of coagulation Thromb Res 2016 145 159 160 27373598 10.1016/j.thromres.2016.06.028

[82] Juang L J Mazinani N Novakowski S K Coagulation factor XII contributes to hemostasis when activated by soil in wounds Blood Adv 2020 4 08 1737 1745 32339233 10.1182/bloodadvances.2019000425 PMC7189277

[83] Mailer R K Rangaswamy C Konrath S Emsley J Renné T An update on factor XII-driven vascular inflammation Biochim Biophys Acta Mol Cell Res 2022 1869 01 119166 34699874 10.1016/j.bbamcr.2021.119166

[84] de Maat S Joseph K Maas C Kaplan A P Blood clotting and the pathogenesis of types I and II hereditary angioedema Clin Rev Allergy Immunol 2021 60 03 348 356 33956309 10.1007/s12016-021-08837-6 PMC8272707

[85] Scheffel J Mahnke N A Hofman Z LM Cold-induced urticarial autoinflammatory syndrome related to factor XII activation Nat Commun 2020 11 01 179 31924766 10.1038/s41467-019-13984-8 PMC6954242

[86] Govers-Riemslag J WP Konings J Cosemans J MEM Impact of deficiency of intrinsic coagulation factors XI and XII on ex vivo thrombus formation and clot lysis TH Open 2019 3 03 e273 e285 31511847 10.1055/s-0039-1693485 PMC6736668

[87] Dickeson S K Kumar S Sun M-F A mechanism for hereditary angioedema caused by a lysine 311-to-glutamic acid substitution in plasminogen Blood 2022 139 18 2816 2829 35100351 10.1182/blood.2021012945 PMC9074402

[88] Stavrou E X Fang C Bane K L Factor XII and uPAR upregulate neutrophil functions to influence wound healing J Clin Invest 2018 128 03 944 959 29376892 10.1172/JCI92880 PMC5824869

[89] Burla F Mulla Y Vos B E From mechanical resilience to active material properties in biopolymer networks Nat Rev Phys 2019 1 249 263

[90] Litvinov R I Weisel J W Fibrin mechanical properties and their structural origins Matrix Biol 2017 60-61 110 123 27553509 10.1016/j.matbio.2016.08.003 PMC5318294

[91] Vos B E Martinez-Torres C Burla F Weisel J W Koenderink G H Revealing the molecular origins of fibrin's elastomeric properties by in situ X-ray scattering Acta Biomater 2020 104 39 52 31923718 10.1016/j.actbio.2020.01.002

[92] Wang Y Kumar S Nisar A Bonn M Rausch M K Parekh S H Probing fibrin's molecular response to shear and tensile deformation with coherent Raman microscopy Acta Biomater 2021 121 383 392 33321217 10.1016/j.actbio.2020.12.020

[93] Undas A Ariëns R AS Fibrin clot structure and function: a role in the pathophysiology of arterial and venous thromboembolic diseases Arterioscler Thromb Vasc Biol 2011 31 12 e88 e99 21836064 10.1161/ATVBAHA.111.230631

[94] Leong L Chernysh I N Xu Y Clot stability as a determinant of effective factor VIII replacement in hemophilia A Res Pract Thromb Haemost 2017 1 02 231 241 29713693 10.1002/rth2.12034 PMC5920517

[95] Wolberg A S Allen G A Monroe D M Hedner U Roberts H R Hoffman M High dose factor VIIa improves clot structure and stability in a model of haemophilia B Br J Haematol 2005 131 05 645 655 16351642 10.1111/j.1365-2141.2005.05820.x

[96] Plodinec M Loparic M Monnier C A The nanomechanical signature of breast cancer Nat Nanotechnol 2012 7 11 757 765 23085644 10.1038/nnano.2012.167

[97] Alkarithi G Duval C Shi Y Macrae F L Ariëns R AS Thrombus structural composition in cardiovascular disease Arterioscler Thromb Vasc Biol 2021 41 09 2370 2383 34261330 10.1161/ATVBAHA.120.315754 PMC8384252

[98] Staessens S François O Brinjikji W Studying stroke thrombus composition after thrombectomy: what can we learn? Stroke 2021 52 11 3718 3727 34517770 10.1161/STROKEAHA.121.034289 PMC8545837

[99] Karbach S H Schönfelder T Brandão I Gut microbiota promote angiotensin II-induced arterial hypertension and vascular dysfunction J Am Heart Assoc 2016 5 09 e003698 27577581 10.1161/JAHA.116.003698 PMC5079031

[100] Han Y-H Onufer E J Huang L-H Enterically derived high-density lipoprotein restrains liver injury through the portal vein Science 2021 373(6553):eabe6729 34437091 10.1126/science.abe6729 PMC8478306

[101] Johnson E L Heaver S L Waters J L Sphingolipids produced by gut bacteria enter host metabolic pathways impacting ceramide levels Nat Commun 2020 11 01 2471 32424203 10.1038/s41467-020-16274-w PMC7235224

[102] Formes H Bernardes J P Mann A The gut microbiota instructs the hepatic endothelial cell transcriptome iScience 2021 24 10 103092 34622147 10.1016/j.isci.2021.103092 PMC8479694

[103] Jäckel S Kiouptsi K Lillich M Gut microbiota regulate hepatic von Willebrand factor synthesis and arterial thrombus formation via Toll-like receptor-2 Blood 2017 130 04 542 553 28572286 10.1182/blood-2016-11-754416

[104] Ascher S Wilms E Pontarollo G Gut microbiota restricts NETosis in acute mesenteric ischemia-reperfusion injury Arterioscler Thromb Vasc Biol 2020 40 09 2279 2292 32611241 10.1161/ATVBAHA.120.314491 PMC7484055

[105] Ascher S Reinhardt C The gut microbiota: an emerging risk factor for cardiovascular and cerebrovascular disease Eur J Immunol 2018 48 04 564 575 29230812 10.1002/eji.201646879

[106] Zhu W Gregory J C Org E Gut microbial metabolite TMAO enhances platelet hyperreactivity and thrombosis risk Cell 2016 165 01 111 124 26972052 10.1016/j.cell.2016.02.011 PMC4862743

[107] Kappel B A De Angelis L Heiser M Cross-omics analysis revealed gut microbiome-related metabolic pathways underlying atherosclerosis development after antibiotics treatment Mol Metab 2020 36 100976 32251665 10.1016/j.molmet.2020.100976 PMC7183232

[108] Stepankova R Tonar Z Bartova J Absence of microbiota (germ-free conditions) accelerates the atherosclerosis in ApoE-deficient mice fed standard low cholesterol diet J Atheroscler Thromb 2010 17 08 796 804 20379054 10.5551/jat.3285

[109] Lindskog Jonsson A Caesar R Akrami R Impact of gut microbiota and diet on the development of atherosclerosis in Apoe ^-/-^ mice Arterioscler Thromb Vasc Biol 2018 38 10 2318 2326 29903735 10.1161/ATVBAHA.118.311233 PMC6166703

[110] Kiouptsi K Jäckel S Pontarollo G The microbiota promotes arterial thrombosis in low-density lipoprotein receptor-deficient mice MBio 2019 10 05 e02298-19 31641089 10.1128/mBio.02298-19 PMC6805995

[111] Kasahara K Krautkramer K A Org E Interactions between Roseburia intestinalis and diet modulate atherogenesis in a murine model Nat Microbiol 2018 3 12 1461 1471 30397344 10.1038/s41564-018-0272-x PMC6280189

[112] Wang Z Klipfell E Bennett B J Gut flora metabolism of phosphatidylcholine promotes cardiovascular disease Nature 2011 472(7341):57 63 21475195 10.1038/nature09922 PMC3086762

[113] Wang Z Roberts A B Buffa J A Non-lethal inhibition of gut microbial trimethylamine production for the treatment of atherosclerosis Cell 2015 163 07 1585 1595 26687352 10.1016/j.cell.2015.11.055 PMC4871610

[114] Haghikia A Li X S Liman T G Gut microbiota-dependent trimethylamine N-oxide predicts risk of cardiovascular events in patients with stroke and is related to proinflammatory monocytes Arterioscler Thromb Vasc Biol 2018 38 09 2225 2235 29976769 10.1161/ATVBAHA.118.311023 PMC6202215

[115] Witkowski M Witkowski M Friebel J Vascular endothelial tissue factor contributes to trimethylamine N-oxide-enhanced arterial thrombosis Cardiovasc Res 2022 118 10 2367 2384 34352109 10.1093/cvr/cvab263 PMC9890461

[116] Schoch L Sutelman P Suades R Badimon L Moreno-Indias I Vilahur G The gut microbiome dysbiosis is recovered by restoring a normal diet in hypercholesterolemic pigs Eur J Clin Invest 2023 53 04 e13927 36453873 10.1111/eci.13927

[117] Tang W HW Bäckhed F Landmesser U Hazen S L Intestinal microbiota in cardiovascular health and disease: JACC State-of-the-Art Review J Am Coll Cardiol 2019 73 16 2089 2105 31023434 10.1016/j.jacc.2019.03.024 PMC6518422

[118] Kiouptsi K Jäckel S Wilms E The commensal microbiota enhances ADP-triggered integrin α _IIb_ β _3_ activation and von Willebrand factor-mediated platelet deposition to type I collagen Int J Mol Sci 2020 21 19 7171 32998468 10.3390/ijms21197171 PMC7583822

[119] Zhang X Zhang X Tong F Gut microbiota induces high platelet response in patients with ST segment elevation myocardial infarction after ticagrelor treatment eLife 2022 11 e70240 35258452 10.7554/eLife.70240 PMC8903831

[120] MetaCardis Consortium Vieira-Silva S Falony G Belda E Statin therapy is associated with lower prevalence of gut microbiota dysbiosis Nature 2020 581(7808):310 315 32433607 10.1038/s41586-020-2269-x

[121] Harper A Vijayakumar V Ouwehand A C Viral infections, the microbiome, and probiotics Front Cell Infect Microbiol 2021 10 596166 33643929 10.3389/fcimb.2020.596166 PMC7907522

[122] Hussain I Cher G LY Abid M A Abid M B Role of gut microbiome in COVID-19: an insight into pathogenesis and therapeutic potential Front Immunol 2021 12 765965 34721437 10.3389/fimmu.2021.765965 PMC8551858

[123] Sun Z Song Z-G Liu C Gut microbiome alterations and gut barrier dysfunction are associated with host immune homeostasis in COVID-19 patients BMC Med 2022 20 01 24 35045853 10.1186/s12916-021-02212-0 PMC8769945

[124] Du X Ley R Buck A H MicroRNAs and extracellular vesicles in the gut: new host modulators of the microbiome? Micro Life 2021 2 10 10.1093/femsml/uqab010 PMC10117826 37223256

[125] Konkoth A Saraswat R Dubrou C Multifaceted role of extracellular vesicles in atherosclerosis Atherosclerosis 2021 319 121 131 33261815 10.1016/j.atherosclerosis.2020.11.006

[126] Zuo T Wu X Wen W Lan P Gut microbiome alterations in COVID-19 Genomics Proteomics Bioinformatics 2021 19 05 679 688 34560321 10.1016/j.gpb.2021.09.004 PMC8478109

[127] Goeijenbier M van Wissen M van de Weg C Review: viral infections and mechanisms of thrombosis and bleeding J Med Virol 2012 84 10 1680 1696 22930518 10.1002/jmv.23354 PMC7166625

[128] Raadsen M Du Toit J Langerak T van Bussel B van Gorp E Goeijenbier M Thrombocytopenia in virus infections J Clin Med 2021 10 04 877 33672766 10.3390/jcm10040877 PMC7924611

[129] Mairuhu A TA Mac Gillavry M R Setiati T E Is clinical outcome of dengue-virus infections influenced by coagulation and fibrinolysis? A critical review of the evidence Lancet Infect Dis 2003 3 01 33 41 12505032 10.1016/s1473-3099(03)00487-0

[130] Goeijenbier M van Gorp E CM Van den Brand J MA Activation of coagulation and tissue fibrin deposition in experimental influenza in ferrets BMC Microbiol 2014 14 134 24884666 10.1186/1471-2180-14-134 PMC4055237

[131] Keller T T Mairuhu A TA de Kruif M D Infections and endothelial cells Cardiovasc Res 2003 60 01 40 48 14522405 10.1016/s0008-6363(03)00354-7

[132] Opal S M Interactions between coagulation and inflammation Scand J Infect Dis 2003 35 09 545 554 14620133 10.1080/00365540310015638

[133] Esmon C T The impact of the inflammatory response on coagulation Thromb Res 2004 114(5–6):321 327 15507261 10.1016/j.thromres.2004.06.028

[134] Levi M van der Poll T Büller H R Bidirectional relation between inflammation and coagulation Circulation 2004 109 22 2698 2704 15184294 10.1161/01.CIR.0000131660.51520.9A

[135] Levi M van der Poll T Schultz M New insights into pathways that determine the link between infection and thrombosis Neth J Med 2012 70 03 114 120 22516575

[136] van der Poll T de Boer J D Levi M The effect of inflammation on coagulation and vice versa Curr Opin Infect Dis 2011 24 03 273 278 21330919 10.1097/QCO.0b013e328344c078

[137] van Gorp E C Suharti C ten Cate H Review: infectious diseases and coagulation disorders J Infect Dis 1999 180 01 176 186 10353876 10.1086/314829

[138] Van Gorp E CM Setiati T E Mairuhu A TA Impaired fibrinolysis in the pathogenesis of dengue hemorrhagic fever J Med Virol 2002 67 04 549 554 12116003 10.1002/jmv.10137

[139] Müller-Calleja N Hollerbach A Royce J Lipid presentation by the protein C receptor links coagulation with autoimmunity Science 2021 371(6534):eabc0956 33707237 10.1126/science.abc0956 PMC9014225

[140] Hollerbach A Müller-Calleja N Pedrosa D Pathogenic lipid-binding antiphospholipid antibodies are associated with severity of COVID-19 J Thromb Haemost 2021 19 09 2335 2347 34242469 10.1111/jth.15455 PMC8420426

[141] Levi M Disseminated intravascular coagulation Crit Care Med 2007 35 09 2191 2195 17855836 10.1097/01.ccm.0000281468.94108.4b

[142] Miller H C Stephan M Hemorrhagic varicella: a case report and review of the complications of varicella in children Am J Emerg Med 1993 11 06 633 638 8240570 10.1016/0735-6757(93)90020-c

[143] Uthman I W Gharavi A E Viral infections and antiphospholipid antibodies Semin Arthritis Rheum 2002 31 04 256 263 11836658 10.1053/sarh.2002.28303

[144] Geisbert T W Jahrling P B Exotic emerging viral diseases: progress and challenges Nat Med 2004 10 12 S110 S121 15577929 10.1038/nm1142

[145] Jing H Wu X Xiang M Liu L Novakovic V A Shi J Pathophysiological mechanisms of thrombosis in acute and long COVID-19 Front Immunol 2022 13 992384 36466841 10.3389/fimmu.2022.992384 PMC9709252

[146] Tanguay J-F Geoffroy P Sirois M G Prevention of in-stent restenosis via reduction of thrombo-inflammatory reactions with recombinant P-selectin glycoprotein ligand-1 Thromb Haemost 2004 91 06 1186 1193 15175806 10.1160/TH03-11-0701

[147] Tomashefski J F Jr Davies P Boggis C Greene R Zapol W M Reid L M The pulmonary vascular lesions of the adult respiratory distress syndrome Am J Pathol 1983 112 01 112 126 6859225 PMC1916312

[148] Jackson S P Darbousset R Schoenwaelder S M Thromboinflammation: challenges of therapeutically targeting coagulation and other host defense mechanisms Blood 2019 133 09 906 918 30642917 10.1182/blood-2018-11-882993

[149] Engelmann B Massberg S Thrombosis as an intravascular effector of innate immunity Nat Rev Immunol 2013 13 01 34 45 23222502 10.1038/nri3345

[150] Ortega-Paz L Talasaz A H Sadeghipour P COVID-19-associated pulmonary embolism: review of the pathophysiology, epidemiology, prevention, diagnosis, and treatment Semin Thromb Hemost 2022(e-pub ahead of print). 10.1055/s-0042-1757634 36223804

[151] Meyers S Crescente M Verhamme P Martinod K *Staphylococcus aureus* and neutrophil extracellular traps: the master manipulator meets its match in immunothrombosis Arterioscler Thromb Vasc Biol 2022 42 03 261 276 35109674 10.1161/ATVBAHA.121.316930 PMC8860219

[152] Iba T Levy J H Inflammation and thrombosis: roles of neutrophils, platelets and endothelial cells and their interactions in thrombus formation during sepsis J Thromb Haemost 2018 16 02 231 241 29193703 10.1111/jth.13911

[153] Gyöngyösi M Alcaide P Asselbergs F W Long COVID and the cardiovascular system - elucidating causes and cellular mechanisms in order to develop targeted diagnostic and therapeutic strategies: a joint Scientific Statement of the ESC Working Groups on Cellular Biology of the Heart and Myocardial & Pericardial Diseases Cardiovasc Res 2023 119 02 336 356 35875883 10.1093/cvr/cvac115 PMC9384470

[154] Subcommittee on Genomics in Thrombosis and Hemostasis Megy K Downes K Simeoni I Curated disease-causing genes for bleeding, thrombotic, and platelet disorders: communication from the SSC of the ISTH J Thromb Haemost 2019 17 08 1253 1260 31179617 10.1111/jth.14479 PMC6852472

[155] Ver Donck F Labarque V Freson K Hemostatic phenotypes and genetic disorders Res Pract Thromb Haemost 2021 5 08 e12637 34964017 10.1002/rth2.12637 PMC8677882

[156] NIHR BioResource Downes K Megy K Duarte D Diagnostic high-throughput sequencing of 2396 patients with bleeding, thrombotic, and platelet disorders Blood 2019 134 23 2082 2091 31064749 10.1182/blood.2018891192 PMC6993014

[157] Megy K Downes K Morel-Kopp M-C GoldVariants, a resource for sharing rare genetic variants detected in bleeding, thrombotic, and platelet disorders: communication from the ISTH SSC Subcommittee on Genomics in Thrombosis and Hemostasis J Thromb Haemost 2021 19 10 2612 2617 34355501 10.1111/jth.15459 PMC9291976

[158] NIHR BioResource for the 100,000 Genomes Project Turro E Astle W J Megy K Whole-genome sequencing of patients with rare diseases in a national health system Nature 2020 583(7814):96 102 32581362 10.1038/s41586-020-2434-2 PMC7610553

[159] BRIDGE-BPD Consortium Westbury S K Turro E Greene D Human phenotype ontology annotation and cluster analysis to unravel genetic defects in 707 cases with unexplained bleeding and platelet disorders Genome Med 2015 7 01 36 25949529 10.1186/s13073-015-0151-5 PMC4422517

[160] Angiolillo D J Capodanno D Danchin N Derivation, validation, and prognostic utility of a prediction rule for nonresponse to clopidogrel: the ABCD-GENE score JACC Cardiovasc Interv 2020 13 05 606 617 32139218 10.1016/j.jcin.2020.01.226

[161] Pinto N Dolan M E Clinically relevant genetic variations in drug metabolizing enzymes Curr Drug Metab 2011 12 05 487 497 21453273 10.2174/138920011795495321 PMC3110519

[162] ESC Scientific Document Group ESC Committee for Practice Guidelines (CPG) ESC National Cardiac Societies Valgimigli M Bueno H Byrne R A 2017 ESC focused update on dual antiplatelet therapy in coronary artery disease developed in collaboration with EACTS: The Task Force for dual antiplatelet therapy in coronary artery disease of the European Society of Cardiology (ESC) and of the European Association for Cardio-Thoracic Surgery (EACTS) Eur Heart J 2018 39 03 213 260 28886622 10.1093/eurheartj/ehx419

[163] TRITON-TIMI 38 Investigators Wiviott S D Braunwald E McCabe C H Prasugrel versus clopidogrel in patients with acute coronary syndromes N Engl J Med 2007 357 20 2001 2015 17982182 10.1056/NEJMoa0706482

[164] PLATO Investigators Wallentin L Becker R C Budaj A Ticagrelor versus clopidogrel in patients with acute coronary syndromes N Engl J Med 2009 361 11 1045 1057 19717846 10.1056/NEJMoa0904327

[165] Vranckx P Leonardi S Tebaldi M Prospective validation of the Bleeding Academic Research Consortium classification in the all-comer PRODIGY trial Eur Heart J 2014 35 37 2524 2529 24755007 10.1093/eurheartj/ehu161

[166] Vranckx P White H D Huang Z Validation of BARC bleeding criteria in patients with acute coronary syndromes: the TRACER trial J Am Coll Cardiol 2016 67 18 2135 2144 27151345 10.1016/j.jacc.2016.02.056

[167] Postula M Janicki P K Rosiak M Effect of common single-nucleotide polymorphisms in acetylsalicylic acid metabolic pathway genes on platelet reactivity in patients with diabetes Med Sci Monit 2013 19 394 408 23715170 10.12659/MSM.883922 PMC3670858

[168] Gurbel P A Rout A Tantry U S Pharmacogenetic considerations in antiplatelet therapy Expert Rev Precis Med Drug Dev 2020 5 235 238

[169] Teng R Butler K Pharmacokinetics, pharmacodynamics, tolerability and safety of single ascending doses of ticagrelor, a reversibly binding oral P2Y(12) receptor antagonist, in healthy subjects Eur J Clin Pharmacol 2010 66 05 487 496 20091161 10.1007/s00228-009-0778-5

[170] Mega J L Close S L Wiviott S D Cytochrome P450 genetic polymorphisms and the response to prasugrel: relationship to pharmacokinetic, pharmacodynamic, and clinical outcomes Circulation 2009 119 19 2553 2560 19414633 10.1161/CIRCULATIONAHA.109.851949

[171] Ancrenaz V Daali Y Fontana P Impact of genetic polymorphisms and drug-drug interactions on clopidogrel and prasugrel response variability Curr Drug Metab 2010 11 08 667 677 20942779 10.2174/138920010794233521

[172] Kazui M Nishiya Y Ishizuka T Identification of the human cytochrome P450 enzymes involved in the two oxidative steps in the bioactivation of clopidogrel to its pharmacologically active metabolite Drug Metab Dispos 2010 38 01 92 99 19812348 10.1124/dmd.109.029132

[173] Mega J L Close S L Wiviott S D Cytochrome p-450 polymorphisms and response to clopidogrel N Engl J Med 2009 360 04 354 362 19106084 10.1056/NEJMoa0809171

[174] Breet N J van Werkum J W Bouman H J Comparison of platelet function tests in predicting clinical outcome in patients undergoing coronary stent implantation JAMA 2010 303 08 754 762 20179285 10.1001/jama.2010.181

[175] Harmsze A van Werkum J W Bouman H J Besides CYP2C19*2, the variant allele CYP2C9*3 is associated with higher on-clopidogrel platelet reactivity in patients on dual antiplatelet therapy undergoing elective coronary stent implantation Pharmacogenet Genomics 2010 20 01 18 25 19934793 10.1097/FPC.0b013e328333dafe

[176] Hulot J-S Bura A Villard E Cytochrome P450 2C19 loss-of-function polymorphism is a major determinant of clopidogrel responsiveness in healthy subjects Blood 2006 108 07 2244 2247 16772608 10.1182/blood-2006-04-013052

[177] Harmsze A M van Werkum J W Ten Berg J M CYP2C19*2 and CYP2C9*3 alleles are associated with stent thrombosis: a case-control study Eur Heart J 2010 31 24 3046 3053 20833683 10.1093/eurheartj/ehq321

[178] Lee C R Luzum J A Sangkuhl K Clinical pharmacogenetics implementation consortium guideline for CYP2C19 genotype and clopidogrel therapy: 2022 update Clin Pharmacol Ther 2022 112 05 959 967 35034351 10.1002/cpt.2526 PMC9287492

[179] Pereira N L Rihal C Lennon R Effect of CYP2C19 genotype on ischemic outcomes during oral P2Y _12_ inhibitor therapy: a meta-analysis JACC Cardiovasc Interv 2021 14 07 739 750 33744207 10.1016/j.jcin.2021.01.024 PMC9853943

[180] Pereira N L Farkouh M E So D Effect of genotype-guided oral P2Y12 inhibitor selection vs conventional clopidogrel therapy on ischemic outcomes after percutaneous coronary intervention: the TAILOR-PCI randomized clinical trial JAMA 2020 324 08 761 771 32840598 10.1001/jama.2020.12443 PMC7448831

[181] ESC Scientific Document Group Collet J-P Thiele H Barbato E 2020 ESC guidelines for the management of acute coronary syndromes in patients presenting without persistent ST-segment elevation Eur Heart J 2021 42 14 1289 1367 32860058 10.1093/eurheartj/ehaa575

[182] Implementing Genomics in Practice (IGNITE) Network Pharmacogenetics Working Group Beitelshees A L Thomas C D Empey P E *CYP2C19* genotype-guided antiplatelet therapy after percutaneous coronary intervention in diverse clinical settings J Am Heart Assoc 2022 11 04 e024159 35156424 10.1161/JAHA.121.024159 PMC9245803

[183] Pan Y Chen W Xu Y Genetic polymorphisms and clopidogrel efficacy for acute ischemic stroke or transient ischemic attack: a systematic review and meta-analysis Circulation 2017 135 01 21 33 27806998 10.1161/CIRCULATIONAHA.116.024913

[184] CHANCE-2 Investigators Wang Y Meng X Wang A Ticagrelor versus clopidogrel in *CYP2C19* loss-of-function carriers with stroke or TIA N Engl J Med 2021 385 27 2520 2530 34708996 10.1056/NEJMoa2111749

[185] Borre E D Goode A Raitz G Predicting thromboembolic and bleeding event risk in patients with non-valvular atrial fibrillation: a systematic review Thromb Haemost 2018 118 12 2171 2187 30376678 10.1055/s-0038-1675400 PMC6754740

[186] Chao T-F Lip G YH Lin Y-J Incident risk factors and major bleeding in patients with atrial fibrillation treated with oral anticoagulants: a comparison of baseline, follow-up and delta HAS-BLED scores with an approach focused on modifiable bleeding risk factors Thromb Haemost 2018 118 04 768 777 29510426 10.1055/s-0038-1636534

[187] Kim H K Tantry U S Smith S C Jr The East Asian Paradox: an updated position statement on the challenges to the current antithrombotic strategy in patients with cardiovascular disease Thromb Haemost 2021 121 04 422 432 33171520 10.1055/s-0040-1718729

[188] Pollack C V Jr Reilly P A van Ryn J Idarucizumab for dabigatran reversal - full cohort analysis N Engl J Med 2017 377 05 431 441 28693366 10.1056/NEJMoa1707278

[189] Lu G DeGuzman F R Hollenbach S J A specific antidote for reversal of anticoagulation by direct and indirect inhibitors of coagulation factor Xa Nat Med 2013 19 04 446 451 23455714 10.1038/nm.3102

[190] Sheffield W P Lambourne M D Eltringham-Smith L J Bhakta V Arnold D M Crowther M A γT -S195A thrombin reduces the anticoagulant effects of dabigatran in vitro and in vivo J Thromb Haemost 2014 12 07 1110 1115 24815541 10.1111/jth.12601

[191] Jourdi G Gouin-Thibault I Siguret V Gandrille S Gaussem P Le Bonniec B FXa-α2-macroglobulin complex neutralizes direct oral anticoagulants targeting FXa in vitro and in vivo Thromb Haemost 2018 118 09 1535 1544 30071567 10.1055/s-0038-1667014

[192] Thalji N K Ivanciu L Davidson R Gimotty P A Krishnaswamy S Camire R M A rapid pro-hemostatic approach to overcome direct oral anticoagulants Nat Med 2016 22 08 924 932 27455511 10.1038/nm.4149 PMC12887842

[193] Parsons-Rich D Hua F Li G Kantaridis C Pittman D D Arkin S Phase 1 dose-escalating study to evaluate the safety, pharmacokinetics, and pharmacodynamics of a recombinant factor Xa variant (FXa ^I16L^ ) J Thromb Haemost 2017 15 05 931 937 28294526 10.1111/jth.13673

[194] Verhoef D Visscher K M Vosmeer C R Engineered factor Xa variants retain procoagulant activity independent of direct factor Xa inhibitors Nat Commun 2017 8 01 528 28904343 10.1038/s41467-017-00647-9 PMC5597622

[195] von Drygalski A Cramer T J Bhat V Griffin J H Gale A J Mosnier L O Improved hemostasis in hemophilia mice by means of an engineered factor Va mutant J Thromb Haemost 2014 12 03 363 372 24818532 10.1111/jth.12489 PMC4161283

[196] von Drygalski A Bhat V Gale A J An engineered factor Va prevents bleeding induced by direct-acting oral anticoagulants by different mechanisms Blood Adv 2020 4 15 3716 3727 32777068 10.1182/bloodadvances.2020001699 PMC7422119

[197] Ansell J Laulicht B E Bakhru S H Ciraparantag, an anticoagulant reversal drug: mechanism of action, pharmacokinetics, and reversal of anticoagulants Blood 2021 137 01 115 125 33205809 10.1182/blood.2020007116

[198] Ansell J Bakhru S Laulicht B E Tracey G Villano S Freedman D Ciraparantag reverses the anticoagulant activity of apixaban and rivaroxaban in healthy elderly subjects Eur Heart J 2022 43 10 985 992 34534272 10.1093/eurheartj/ehab637 PMC8900496

[199] Caruso C Lam W A Point-of-care diagnostic assays and novel preclinical technologies for hemostasis and thrombosis Semin Thromb Hemost 2021 47 02 120 128 33636744 10.1055/s-0041-1723798

[200] Boender J Kruip M JHA Leebeek F WG A diagnostic approach to mild bleeding disorders J Thromb Haemost 2016 14 08 1507 1516 27208505 10.1111/jth.13368

[201] Moenen F CJI Vries M JA Nelemans P J Screening for platelet function disorders with Multiplate and platelet function analyzer Platelets 2019 30 01 81 87 29135309 10.1080/09537104.2017.1371290

[202] Panova-Noeva M van der Meijden P EJ Ten Cate H Clinical applications, pitfalls, and uncertainties of thrombin generation in the presence of platelets J Clin Med 2019 9 01 92 31905839 10.3390/jcm9010092 PMC7019916

[203] Branchford B R Ng C J Neeves K B Di Paola J Microfluidic technology as an emerging clinical tool to evaluate thrombosis and hemostasis Thromb Res 2015 136 01 13 19 26014643 10.1016/j.thromres.2015.05.012 PMC4910695

[204] de Breet C PDM Zwaveling S Vries M JA Thrombin generation as a method to identify the risk of bleeding in high clinical-risk patients using dual antiplatelet therapy Front Cardiovasc Med 2021 8 679934 34179143 10.3389/fcvm.2021.679934 PMC8224526

[205] Hulshof A-M Olie R H Vries M JA Rotational thromboelastometry in high-risk patients on dual antithrombotic therapy after percutaneous coronary intervention Front Cardiovasc Med 2021 8 788137 35004899 10.3389/fcvm.2021.788137 PMC8727359

[206] Brouns S LN van Geffen J P Heemskerk J WM High-throughput measurement of human platelet aggregation under flow: application in hemostasis and beyond Platelets 2018 29 07 662 669 29537929 10.1080/09537104.2018.1447660

[207] Qiu Y Ahn B Sakurai Y Microvasculature-on-a-chip for the long-term study of endothelial barrier dysfunction and microvascular obstruction in disease Nat Biomed Eng 2018 2 453 463 30533277 10.1038/s41551-018-0224-z PMC6286070

[208] Sakurai Y Hardy E T Ahn B A microengineered vascularized bleeding model that integrates the principal components of hemostasis Nat Commun 2018 9 01 509 29410404 10.1038/s41467-018-02990-x PMC5802762

[209] Subcommittee on Biorheology Mangin P H Gardiner E E Nesbitt W S In vitro flow based systems to study platelet function and thrombus formation: recommendations for standardization: communication from the SSC on Biorheology of the ISTH J Thromb Haemost 2020 18 03 748 752 32112535 10.1111/jth.14717

[210] Sniderman J Monagle P Annich G M MacLaren G Hematologic concerns in extracorporeal membrane oxygenation Res Pract Thromb Haemost 2020 4 04 455 468 32548547 10.1002/rth2.12346 PMC7292669

[211] Olson S R Murphree C R Zonies D Thrombosis and bleeding in extracorporeal membrane oxygenation (ECMO) without anticoagulation: a systematic review ASAIO J 2021 67 03 290 296 33627603 10.1097/MAT.0000000000001230 PMC8623470

[212] Vaquer S de Haro C Peruga P Oliva J C Artigas A Systematic review and meta-analysis of complications and mortality of veno-venous extracorporeal membrane oxygenation for refractory acute respiratory distress syndrome Ann Intensive Care 2017 7 01 51 28500585 10.1186/s13613-017-0275-4 PMC5429319

[213] Noah M A Peek G J Finney S J Referral to an extracorporeal membrane oxygenation center and mortality among patients with severe 2009 influenza A(H1N1) JAMA 2011 306 15 1659 1668 21976615 10.1001/jama.2011.1471

[214] REVA Research Network Pham T Combes A Rozé H Extracorporeal membrane oxygenation for pandemic influenza A(H1N1)-induced acute respiratory distress syndrome: a cohort study and propensity-matched analysis Am J Respir Crit Care Med 2013 187 03 276 285 23155145 10.1164/rccm.201205-0815OC

[215] Aubron C DePuydt J Belon F Predictive factors of bleeding events in adults undergoing extracorporeal membrane oxygenation Ann Intensive Care 2016 6 01 97 27714705 10.1186/s13613-016-0196-7 PMC5053950

[216] Brogan T V Thiagarajan R R Rycus P T Bartlett R H Bratton S L Extracorporeal membrane oxygenation in adults with severe respiratory failure: a multi-center database Intensive Care Med 2009 35 12 2105 2114 19768656 10.1007/s00134-009-1661-7

[217] Aubron C Cheng A C Pilcher D Factors associated with outcomes of patients on extracorporeal membrane oxygenation support: a 5-year cohort study Crit Care 2013 17 02 R73 23594433 10.1186/cc12681 PMC4056036

[218] Murphy D A Hockings L E Andrews R K Extracorporeal membrane oxygenation-hemostatic complications Transfus Med Rev 2015 29 02 90 101 25595476 10.1016/j.tmrv.2014.12.001

[219] Nguyen T P Phan X T Nguyen T H Major bleeding in adults undergoing peripheral extracorporeal membrane oxygenation (ECMO): prognosis and predictors Crit Care Res Pract 2022 2022 5.348835E6 10.1155/2022/5348835 PMC8783736 35075397

[220] Cashen K Meert K Dalton H Anticoagulation in neonatal ECMO: an enigma despite a lot of effort! Front Pediatr 2019 7 366 31572699 10.3389/fped.2019.00366 PMC6753198

[221] Hunt B J Parratt R N Segal H C Sheikh S Kallis P Yacoub M Activation of coagulation and fibrinolysis during cardiothoracic operations Ann Thorac Surg 1998 65 03 712 718 9527200 10.1016/s0003-4975(97)01345-3

[222] Heilmann C Geisen U Beyersdorf F Acquired von Willebrand syndrome in patients with extracorporeal life support (ECLS) Intensive Care Med 2012 38 01 62 68 21965100 10.1007/s00134-011-2370-6

[223] Palatianos G M Foroulis C N Vassili M I A prospective, double-blind study on the efficacy of the bioline surface-heparinized extracorporeal perfusion circuit Ann Thorac Surg 2003 76 01 129 135 12842526 10.1016/s0003-4975(03)00338-2

[224] Bembea M M Annich G Rycus P Oldenburg G Berkowitz I Pronovost P Variability in anticoagulation management of patients on extracorporeal membrane oxygenation: an international survey Pediatr Crit Care Med 2013 14 02 e77 e84 23287906 10.1097/PCC.0b013e31827127e4 PMC3567253

[225] ELSO Registry Paden M L Conrad S A Rycus P T Thiagarajan R R Extracorporeal Life Support Organization Registry Report 2012 ASAIO J 2013 59 03 202 210 23644605 10.1097/MAT.0b013e3182904a52

[226] Kim J Koo B-K Knoblich J A Human organoids: model systems for human biology and medicine Nat Rev Mol Cell Biol 2020 21 10 571 584 32636524 10.1038/s41580-020-0259-3 PMC7339799

[227] Human Cell Atlas ‘Biological Network’ Organoids Bock C Boutros M Camp J G The organoid cell atlas Nat Biotechnol 2021 39 01 13 17 33384458 10.1038/s41587-020-00762-x PMC7801253

[228] Veninga V Voest E E Tumor organoids: opportunities and challenges to guide precision medicine Cancer Cell 2021 39 09 1190 1201 34416168 10.1016/j.ccell.2021.07.020

[229] Khan A O Reyat J S Hill H Preferential uptake of SARS-CoV-2 by pericytes potentiates vascular damage and permeability in an organoid model of the microvasculature Cardiovasc Res 2022 118 15 3085 3096 35709328 10.1093/cvr/cvac097 PMC9214165

[230] Khan A O Colombo M Reyat J S Human bone marrow organoids for disease modelling, discovery and validation of therapeutic targets in hematological malignanciesbioRxiv 2022. Accessed September 11, 2022 at:https://www.biorxiv.org/content/biorxiv/early/2022/03/16/2022.03.14.483815 10.1158/2159-8290.CD-22-0199 PMC9900323 36351055

[231] Campello E Spiezia L Adamo A Simioni P Thrombophilia, risk factors and prevention Expert Rev Hematol 2019 12 03 147 158 30773075 10.1080/17474086.2019.1583555

[232] Castoldi E Hézard N Mourey G Severe thrombophilia in a factor V-deficient patient homozygous for the Ala2086Asp mutation (FV Besançon) J Thromb Haemost 2021 19 05 1186 1199 33605529 10.1111/jth.15274

[233] Simioni P Castoldi E Lunghi B Tormene D Rosing J Bernardi F An underestimated combination of opposites resulting in enhanced thrombotic tendency Blood 2005 106 07 2363 2365 15961511 10.1182/blood-2005-04-1461

[234] Simioni P Tormene D Tognin G X-linked thrombophilia with a mutant factor IX (factor IX Padua) N Engl J Med 2009 361 17 1671 1675 19846852 10.1056/NEJMoa0904377

[235] Wu W Xiao L Wu X Factor IX alteration p.Arg338Gln (FIX Shanghai) potentiates FIX clotting activity and causes thrombosis Haematologica 2021 106 01 264 268 32079698 10.3324/haematol.2019.216713 PMC7776343

[236] Simioni P Cagnin S Sartorello F Partial F8 gene duplication (factor VIII Padua) associated with high factor VIII levels and familial thrombophilia Blood 2021 137 17 2383 2393 33275657 10.1182/blood.2020008168

[237] Takagi Y Murata M Kozuka T Missense mutations in the gene encoding prothrombin corresponding to Arg596 cause antithrombin resistance and thrombomodulin resistance Thromb Haemost 2016 116 06 1022 1031 27604259 10.1160/TH16-03-0223

[238] Djordjevic V Kovac M Miljic P A novel prothrombin mutation in two families with prominent thrombophilia – the first cases of antithrombin resistance in a Caucasian population J Thromb Haemost 2013 11 10 1936 1939 23927452 10.1111/jth.12367

[239] Sivasundar S Oommen A T Prakash O Molecular defect of ‘Prothrombin Amrita’: substitution of arginine by glutamine (Arg553 to Gln) near the Na(+) binding loop of prothrombin Blood Cells Mol Dis 2013 50 03 182 183 23265743 10.1016/j.bcmd.2012.11.008

[240] Bulato C Radu C M Campello E New prothrombin mutation (Arg596Trp, Prothrombin Padua 2) associated with venous thromboembolism Arterioscler Thromb Vasc Biol 2016 36 05 1022 1029 27013614 10.1161/ATVBAHA.115.306914

[241] Tuinenburg A Mauser-Bunschoten E P Verhaar M C Biesma D H Schutgens R E Cardiovascular disease in patients with hemophilia J Thromb Haemost 2009 7 02 247 254 18983484 10.1111/j.1538-7836.2008.03201.x

[242] ADVANCE working group Schutgens R EG Klamroth R Pabinger I Dolan G Management of atrial fibrillation in people with haemophilia–a consensus view by the ADVANCE Working Group Haemophilia 2014 20 06 e417 e420 25296569 10.1111/hae.12525

[243] Martin K Key N S How I treat patients with inherited bleeding disorders who need anticoagulant therapy Blood 2016 128 02 178 184 27106121 10.1182/blood-2015-12-635094 PMC4946199

[244] Ferraris V A Boral L I Cohen A J Smyth S S White G C II Consensus review of the treatment of cardiovascular disease in people with hemophilia A and B Cardiol Rev 2015 23 02 53 68 25436468 10.1097/CRD.0000000000000045 PMC4323575

[245] Shapiro S Makris M Haemophilia and ageing Br J Haematol 2019 184 05 712 720 30592034 10.1111/bjh.15745

[246] Guillet B Cayla G Lebreton A Long-term antithrombotic treatments prescribed for cardiovascular diseases in patients with hemophilia: results from the French registry Thromb Haemost 2021 121 03 287 296 33099283 10.1055/s-0040-1718410 PMC7895544

[247] de Koning M LY Fischer K de Laat B Huisman A Ninivaggi M Schutgens R EG Comparing thrombin generation in patients with hemophilia A and patients on vitamin K antagonists J Thromb Haemost 2017 15 05 868 875 28296129 10.1111/jth.13674

[248] van den Ham H A Souverein P C Klungel O H Major bleeding in users of direct oral anticoagulants in atrial fibrillation: a pooled analysis of results from multiple population-based cohort studies Pharmacoepidemiol Drug Saf 2021 30 10 1339 1352 34173286 10.1002/pds.5317 PMC8456818

[249] Nagy M Perrella G Dalby A Flow studies on human GPVI-deficient blood under coagulating and noncoagulating conditions Blood Adv 2020 4 13 2953 2961 32603422 10.1182/bloodadvances.2020001761 PMC7362345

[250] Voors-Pette C Lebozec K Dogterom P Safety and tolerability, pharmacokinetics, and pharmacodynamics of ACT017, an antiplatelet GPVI (Glycoprotein VI) Fab Arterioscler Thromb Vasc Biol 2019 39 05 956 964 31017822 10.1161/ATVBAHA.118.312314

[251] Cooper N Altomare I Thomas M R Assessment of thrombotic risk during long-term treatment of immune thrombocytopenia with fostamatinib Ther Adv Hematol 2021 12 20406207211010875 10.1177/20406207211010875 PMC8111531 33995988

[252] Rayes J Watson S P Nieswandt B Functional significance of the platelet immune receptors GPVI and CLEC-2 J Clin Invest 2019 129 01 12 23 30601137 10.1172/JCI122955 PMC6307936

[253] Nicolson P L Welsh J D Chauhan A Thomas M R Kahn M L Watson S P A rationale for blocking thromboinflammation in COVID-19 with Btk inhibitors Platelets 2020 31 05 685 690 32552307 10.1080/09537104.2020.1775189

[254] Toh C-H Wang G Parker A L The aetiopathogenesis of vaccine-induced immune thrombotic thrombocytopenia Clin Med (Lond) 2022 22 02 140 144 35273026 10.7861/clinmed.2022-0006 PMC8966812

[255] Smith C W Montague S J Kardeby C Antiplatelet drugs block platelet activation by VITT patient serum Blood 2021 138 25 2733 2740 34375398 10.1182/blood.2021012277 PMC8697531

[256] Nicolson P LR Nock S H Hinds J Low-dose Btk inhibitors selectively block platelet activation by CLEC-2 Haematologica 2021 106 01 208 219 31949019 10.3324/haematol.2019.218545 PMC7776357

[257] Tillman B F Gruber A McCarty O JT Gailani D Plasma contact factors as therapeutic targets Blood Rev 2018 32 06 433 448 30075986 10.1016/j.blre.2018.04.001 PMC6185818

[258] Zhang H Löwenberg E C Crosby J R Inhibition of the intrinsic coagulation pathway factor XI by antisense oligonucleotides: a novel antithrombotic strategy with lowered bleeding risk Blood 2010 116 22 4684 4692 20807891 10.1182/blood-2010-04-277798

[259] Cheng Q Tucker E I Pine M S A role for factor XIIa-mediated factor XI activation in thrombus formation in vivo Blood 2010 116 19 3981 3989 20634381 10.1182/blood-2010-02-270918 PMC2981546

[260] FXI-ASO TKA Investigators Büller H R Bethune C Bhanot S Factor XI antisense oligonucleotide for prevention of venous thrombosis N Engl J Med 2015 372 03 232 240 25482425 10.1056/NEJMoa1405760 PMC4367537

[261] ANT-005 TKA Investigators Verhamme P Yi B A Segers A Abelacimab for prevention of venous thromboembolism N Engl J Med 2021 385 07 609 617 34297496 10.1056/NEJMoa2105872

[262] AXIOMATIC-TKR Investigators Weitz J I Strony J Ageno W Milvexian for the prevention of venous thromboembolism N Engl J Med 2021 385 23 2161 2172 34780683 10.1056/NEJMoa2113194 PMC9540352

[263] Liuzzo G Patrono C A novel inhibitor of factor XIa as potential haemostasis-sparing anticoagulant for patients with atrial fibrillation Eur Heart J 2022 43 25 2354 2355 35551379 10.1093/eurheartj/ehac250

[264] PACIFIC AMI Investigators Rao S V Kirsch B Bhatt D L A multicenter, phase 2, randomized, placebo-controlled, double-blind, parallel-group, dose-finding trial of the oral factor XIa inhibitor asundexian to prevent adverse cardiovascular outcomes after acute myocardial infarction Circulation 2022 146 16 1196 1206 36030390 10.1161/CIRCULATIONAHA.122.061612

[265] PACIFIC-Stroke Investigators Shoamanesh A Mundl H Smith E E Factor XIa inhibition with asundexian after acute non-cardioembolic ischaemic stroke (PACIFIC-Stroke): an international, randomised, double-blind, placebo-controlled, phase 2b trial Lancet 2022 400(10357):997 1007 36063821 10.1016/S0140-6736(22)01588-4

[266] Thachil J Lessons learnt from COVID-19 coagulopathy eJHaem 2021 2 03 577 584 34226900 10.1002/jha2.228 PMC8242569

[267] Levi M Thachil J Iba T Levy J H Coagulation abnormalities and thrombosis in patients with COVID-19 Lancet Haematol 2020 7 06 e438 e440 32407672 10.1016/S2352-3026(20)30145-9 PMC7213964

[268] Thachil J Srivastava A SARS-2 coronavirus-associated hemostatic lung abnormality in COVID-19: is it pulmonary thrombosis or pulmonary embolism? Semin Thromb Hemost 2020 46 07 777 780 32396963 10.1055/s-0040-1712155 PMC7645824

[269] Thachil J All those D-dimers in COVID-19 J Thromb Haemost 2020 18 08 2075 2076 32470213 10.1111/jth.14939 PMC7283856

[270] Thachil J What do monitoring platelet counts in COVID-19 teach us? J Thromb Haemost 2020 18 08 2071 2072 32344467 10.1111/jth.14879 PMC7267313

[271] Thachil J Tang N Gando S ISTH interim guidance on recognition and management of coagulopathy in COVID-19 J Thromb Haemost 2020 18 05 1023 1026 32338827 10.1111/jth.14810 PMC9906133

[272] Lacroix R Sabatier F Mialhe A Activation of plasminogen into plasmin at the surface of endothelial microparticles: a mechanism that modulates angiogenic properties of endothelial progenitor cells in vitro Blood 2007 110 07 2432 2439 17606760 10.1182/blood-2007-02-069997 PMC2495018

[273] Dejouvencel T Doeuvre L Lacroix R Fibrinolytic cross-talk: a new mechanism for plasmin formation Blood 2010 115 10 2048 2056 19996088 10.1182/blood-2009-06-228817 PMC2896557

[274] CointeSVallierLEsnaultPGranulocyte microvesicles with a high plasmin generation capacity promote clot lysis and improve outcome in septic shockBlood202213915237723913502600410.1182/blood.2021013328

[275] Lacroix R Plawinski L Robert S Leukocyte- and endothelial-derived microparticles: a circulating source for fibrinolysis Haematologica 2012 97 12 1864 1872 22733025 10.3324/haematol.2012.066167 PMC3590093

[276] Lacroix R Dubois C Leroyer A S Sabatier F Dignat-George F Revisited role of microparticles in arterial and venous thrombosis J Thromb Haemost 2013 11 01 24 35 23809108 10.1111/jth.12268

[277] Vallier L Cointe S Lacroix R Microparticles and fibrinolysis Semin Thromb Hemost 2017 43 02 129 134 27923263 10.1055/s-0036-1592301

[278] Cointe S Harti Souab K Bouriche T A new assay to evaluate microvesicle plasmin generation capacity: validation in disease with fibrinolysis imbalance J Extracell Vesicles 2018 7 01 1.494482E6 10.1080/20013078.2018.1494482 PMC6052415 30034644

[279] Vallier L Bouriche T Bonifay A Increasing the sensitivity of the human microvesicle tissue factor activity assay Thromb Res 2019 182 64 74 31450010 10.1016/j.thromres.2019.07.011 PMC6825876

[280] Franco C Lacroix R Vallier L A new hybrid immunocapture bioassay with improved reproducibility to measure tissue factor-dependent procoagulant activity of microvesicles from body fluids Thromb Res 2020 196 414 424 33038585 10.1016/j.thromres.2020.09.020

